# GC-MS Profile, Antioxidant Activity, and In Silico Study of the Essential Oil from *Schinus molle* L. Leaves in the Presence of Mosquito Juvenile Hormone-Binding Protein (mJHBP) from *Aedes aegypti*

**DOI:** 10.1155/2022/5601531

**Published:** 2022-05-16

**Authors:** Oscar Herrera-Calderon, Haydee Chavez, Edwin Carlos Enciso-Roca, Pablo Williams Común-Ventura, Renan Dilton Hañari-Quispe, Linder Figueroa-Salvador, Eddie Loyola-Gonzales, Josefa Bertha Pari-Olarte, Nada H. Aljarba, Saad Alkahtani, Gaber El-Saber Batiha

**Affiliations:** ^1^Department of Pharmacology, Bromatology and Toxicology, Faculty of Pharmacy and Biochemistry, Universidad Nacional Mayor de San Marcos, Lima, Peru; ^2^Department of Pharmaceutical Chemistry, Faculty of Pharmacy and Biochemistry, Universidad Nacional San Luis Gonzaga, Ica 11001, Peru; ^3^Department of Human Medicine, Faculty of Health Sciences, Universidad Nacional de San Cristobal de Huamanga, Portal Independencia 57, Ayacucho 05003, Peru; ^4^Clinical Pathology Laboratory, Faculty of Veterinary Medicine and Zootechnics, Universidad Nacional del Altiplano, Av Floral 1153, Puno 21001, Peru; ^5^School of Medicine, Faculty of Health Sciences, Universidad Peruana de Ciencias Aplicadas, Prolongación Primavera 2390, Lima 15023, Peru; ^6^Department of Pharmaceutical Science, Faculty of Pharmacy and Biochemistry, Universidad Nacional San Luis Gonzaga, Ica 11001, Peru; ^7^Department of Biology, College of Science, Princess Nourah bint Abdulrahman University, P. O. Box 84428, Riyadh 11671, Saudi Arabia; ^8^Department of Zoology, College of Science, King Saud University, P. O. Box 2455, Riyadh 11451, Saudi Arabia; ^9^Department of Pharmacology and Therapeutics, Faculty of Veterinary Medicine, Damanhour University, Damanhur, 22511 El-Beheira, Egypt

## Abstract

*Schinus molle* is a medicinal plant used as an anti-inflammatory and for rheumatic pain in the traditional medicine of Peru. On the other hand, *Aedes aegypti* is the main vector of several tropical diseases and the transmitter of yellow fever, chikungunya, malaria, dengue, and Zika virus. In this study, the aim was to investigate the antioxidant activity in vitro and the insecticidal activity in silico, in the presence of the mosquito juvenile hormone-binding protein (mJHBP) from *Aedes aegypti*, of the essential oil from *S. molle* leaves. The volatile phytochemicals were analyzed by gas chromatography-mass spectrometry (GC-MS), and the profile antioxidants were examined by DPPH, ABTS, and FRAP assays. The evaluation in silico was carried out on mJHBP (PDB: 5V13) with an insecticidal approach. The results revealed that EO presented as the main volatile components to alpha-phellandrene (32.68%), D-limonene (12.59%), and beta-phellandrene (12.24%). The antioxidant activity showed values for DPPH = 11.42 ± 0.08 *μ*mol ET/g, ABTS = 134.88 ± 4.37 *μ*mol ET/g, and FRAP = 65.16 ± 1.46 *μ*mol ET/g. Regarding the insecticidal approach in silico, alpha-muurolene and gamma-cadinene had the best biding energy on mJHBP (Δ*G* = −9.7 kcal/mol), followed by beta-cadinene (Δ*G* = −9.5 kcal/mol). Additionally, the volatile components did not reveal antioxidant activity, and its potential insecticidal effect would be acting on mJHBP from *A. aegypti.*

## 1. Introduction

Essential oils (EOs) are a class of natural products characterized by their volatile components, especially terpenes and sesquiterpenes [1]. EOs are obtained by several methods such as hydrodistillation, supercritical fluids, microwaves, and ultrasound [2]. Although the yield rate is very low, a majority are used in perfumery [3], aromatherapy [4], and the pharmaceutical [5] and food industries [6]. Their biological activities have been reported as insecticidal, antiviral, antibacterial, antifungal, antimalarial, antidepressive, anticancer, antimutagenic, hepatoprotective, anti-inflammatory, antioxidant, analgesic, and antipyretic [5, 7]. *Schinus molle* L., internationally commonly known as peruvian pepper, false pepper, American pepper, or pink pepper, is a perennial tree belonging to the Anacardiaceae family, which is native to subtropical regions of South and Central America [8]. In Peru, it is popularly known as molle (in Spanish) and its leaves and fruits exhibit anti-inflammatory, analgesic, antirheumatic, antibacterial, antiseptic, and repellent activity [9]. The essential oil of *S. molle* (leaves and fruits) exhibits biological activities such as antibacterial (gram-positive and gram-negative bacteria), antifungal, insecticidal, repellent, and cytotoxic activities [10]. Locally, the plant is used as a rheumatic reliever, analgesic, antiseptic, purgative, stomach cramp reliever, and diuretic. Moreover, leaves, fruits, and latex are used for many conditions, including menstrual, respiratory, and urinary disorders, and as an antidepressant, digestive stimulant, and astringent [11].

Regarding insecticidal activity, several synthetized chemicals are used as repellents, vector control, or insecticides, but during the last year, some of them have been retired due to their carcinogenic, genotoxic, or harmful-to-the-environment effects by bioaccumulation [12]. The application of natural products as insecticides might be highly effective, less expensive, biodegradable, and safer than that of synthetic insecticides [13]. The harmful effect of essential oils or their isolated compounds against insects can be manifested in various ways, including mortality, toxicity, inhibiting growth, the suppression of reproductive behavior, and reducing fertility and fecundity [14]. Hence, the use of bioinsecticides based on natural products might be a powerful tool to combat some insects who are responsible for several tropical diseases (Zika, malaria, dengue, and yellow fever) transmitted mainly by the mosquito *Aedes aegypti* [15].

Recently, a group of salivary D7 proteins known as mosquito juvenile hormone-binding protein (mJHBP) has been identified in *A. aegypti*, which consist of two modified odorant-binding protein domains with its respective ligand in the N-terminal domain named JH-III [16]. This hormone is found in pupae and adults; furthermore, it regulates the larval development in the mosquito. On the other hand, the juvenile hormone (JH) plays an essential role in adult female promoting the ovary maturation before blood feeding; additionally, it is involved with the nutritional state and its relationship with blood meal-dependent reproductive development [17]. In recent years, a class of insecticides named JH analogues have been designed to disrupt this endocrine process and affect the normal development in mosquitoes. A synthetic JH analogue is pyriproxyfen, which is a phenyl carbonyl derivative, and its main effect is to produce an imbalance in the mosquito hormonal system, inhibiting the embryogenesis, adult metamorphosis, and development of the adult mosquito [18].

Although the insecticidal activity of *S. molle* EO was evaluated in several species such as *Trogoderma granarium*, *Tribolium castaneum* [19], *Sitophilus oryzae* [20], *Haematobia irritans* [21], *Ctenocephalides felis felis* [22], and *Gonipterus platensis* [23], currently, there is not scientific literature on *A. aegypti*. Thus, we investigated the potential insecticidal effect in silico of the volatile components of *S. molle* EO to find any responsible molecule as a JH analogue, which might combat the presence of mosquitoes in tropical regions and reduce the prevalence of diseases transmitted by them. The aim in this study was (1) to determine the total volatile component of the EO from *S. molle* leaves by gas chromatography-mass spectrometry (GC-MS), (2) to evaluate the antioxidant activity using the DPPH, ABTS, and FRAP methods, and (3) to determine the insecticidal activity using a virtual screening of the EO from *S. molle* leaves on the mosquito juvenile hormone-binding protein from *Aedes aegypti.*

## 2. Materials and Methods

### 2.1. Chemicals

All the solvents (dichloromethane, chloroform, methanol, and hydrogen peroxide), of analytical grade (99.5%), were purchased from Merck (Darmstadt, Germany). 2,2-Diphenyl-1-picrylhydrazyl (DPPH), 2,2′-azino-bis(3-ethylbenzothiazoline-6-sulfonic acid) (ABTS), 2,4,6-tri(2-pyridyl)-s-triazine (TPTZ), and Trolox were purchased from Sigma-Aldrich (St. Louis, MO, USA).

### 2.2. Plant Material

A quantity of 4700 g of *Schinus molle* (leaves) cultivated in Tinguiña, Ica Region, Peru (406 m.a.s.l.) in December 2020 was received. Leaves were cleaned and peeled to be incorporated in a Clevenger equipment and to obtain essential oil by hydrodistillation for 2 h [24]. The essential oil was separated by decantation; then, anhydrous Na_2_SO_4_ was added to eliminate any water drops. Finally, the EO was stored in a sealed amber vial until further use.

### 2.3. Identification of Volatile Compounds by Gas Chromatography-Mass Spectrometry (GC-MS)

Volatile chemicals were determined with a GC-MS system (Agilent Technologies 7890 Gas Detector and Agilent Technologies 5975C Mass Spectrometer Detector, Santa Clara, CA, USA). Then, 20 *μ*L of EO was mixed with 1.0 mL of dichloromethane. Next, 1.0 *μ*L of the working solution was injected into the equipment in splitless mode (split: 20 : 1). The EO was run on a J&W 122-1545.67659 DB-5 ms column, (60 m × 250 *μ*m × 0.25 *μ*m) (Agilent Technologies, Santa Clara, CA, USA). The working conditions were as follows: the temperature program was 40°C, starting with increments of 5°C/min up to 180°C, followed by increases of 2.5°C/min up to 200°C for 5 min, and finally 10°C/min up to 300°C, remaining for 3 min. The helium flow rate was at 1 mL/min. Volatile components' identification was based on a comparison of relative retention indices (RIs) and mass spectra data with the NIST20 library data and the published literature [25]. Each RI was calculated compared with a homologous series of n-alkanes C9–C25 (C9, BHD purity 99%; C10–C25, Fluka purity 99%). The relative amount (expressed as a percentage) of each compound identified in the EO was calculated by comparing the area of the corresponding peak in the chromatogram with the total area of identified peaks.

### 2.4. Determination of the Antioxidant Capacity by the Free Radical 2,2-Diphenyl-1-picrylhydrazyl (DPPH)

A 150 *μ*L of EO (10 mg/mL) was mixed with 2850 *μ*L of a methanolic solution of DPPH (20 mg/L) with an absorbance adjusted to 1.1 ± 0.02 nm. After mixing, it was incubated in the dark for 30 minutes, and the absorbance reading was carried out at 515 nm. The standard curve was elaborated with Trolox at concentrations of 0 to 800 *μ*mol/mL. The Trolox equivalent antioxidant capacity (TEAC) was expressed as *μ*mol TE/g of essential oil [26]. To calculate the half inhibitory concentration (IC50), a linear regression method was used based on the different concentrations of the EO.

### 2.5. Determination of the Antioxidant Capacity by the Method of the Radical 2,2′-Azinobis-(3-ethylbenzothiazoline)-6-sulfonic Acid (ABTS^.+^)

To carry out the antioxidant activity using the ABTS radical, a solution was prepared using the mixing of 10 mL of ABTS (4.06 mg/mL) and 10 mL of potassium persulfate (0.7 mg/mL), both reacted for 16 hours. Then, 150 *μ*L of the EO (5 mg/mL, diluted with methanol) was mixed with 2850 *μ*L of ABTS radical and incubated for 7 min, the same procedure was used with Trolox standard ranging between 0 and 400 *μ*mol/mL. The absorbance was read to 0.7 ± 0.02 at a wavelength of 734 nm. The Trolox equivalent antioxidant capacity (TEAC) was expressed as *μ*mol TE/g of essential oil [27]. To calculate the half inhibitory concentration (IC_50_), a linear regression method was used based on the different concentrations of the EO.

### 2.6. Determination of Antioxidant Capacity by the Ferric Reducing/Antioxidant Power (FRAP)

In the determination of FRAP of EO, a reagent battery was made using 25 mL of acetate buffer pH 3.6; 2.5 mL of 20 mM TPTZ was dissolved in 40 mM HCl and 2.5 mL of 20 mM ferric chloride hexahydrate. The mixing of this reagents constituted the FRAP solution which reacted with the EO at different concentrations as well as Trolox standard. 150 *μ*L of EO (1 mg/mL) was mixed with 2850 *μ*L of FRAP reagent and reacted for 4 min at room temperature. The absorbance reading was carried out at 593 nm. A standard curve was prepared with Trolox (50-800 *μ*M). The results were expressed as *μ*mol equivalents of Trolox per gram of essential oil (*μ*mol TE/g EO) [28]. To calculate the half inhibitory concentration (IC_50_), the linear regression method was used based on the different concentrations of the EO.

### 2.7. Molecular Docking Studies

The molecules isolated from phytochemicals of essential oil were docked against the mosquito juvenile hormone-binding protein (PDB id: 5V13). In order to validate the docking, the crystal structures were docked with the native ligand pyriproxifen and JH3 bound to X-ray structures of mosquito juvenile hormone-binding protein. In addition to the former for comparison, better validation of docking at the binding cavity of mosquito juvenile hormone-binding protein was determined surrounding the 3 Å distance from the bound ligand JH3 using the DoGSiteScorer server. The binding cavity residues comprised mainly TYR33, LEU37, TRP50, AL51, TRP53, TYR64, SER69, TYR129, TYR133, ILE140, PHE269, TRP278, and ALA281. Protein and ligand preparations were performed using AutoDock Tools (v. 1.5.6) (Forli et al., 2016). Gasteiger charges were added to the ligand molecules prior to converting to PDBQT format. The online server DoGSiteScorer and the information about the binding site residues of the native ligand were used to construct the grid box. The grid box of dimensions 25 × 25 × 14 Å for the mosquito juvenile hormone-binding protein with 0.375 Å grid spacing was constructed using AutoGrid 4.2. Semiflexible docking was performed keeping the receptor molecule rigid and ligands flexible. AutoDock 4.2 using the Lamarckian genetic algorithm (LGA) scoring function with number of GA runs = 100, population size = 500, and maximum number of evaluations = 25,000,000 was used to develop the molecular docking of all volatile components of EO. Then, the RMSD clustering maps were obtained by a reclustering command with a clustering tolerance of 0.25 Å, 0.5 Å, and 1 Å, respectively, in order to obtain the best cluster having the lowest energy score with a high number of populations [18].

### 2.8. Molecular Dynamics Simulation (MD)

This evaluation was carried on the dock complexes for *α*-phellandrene (the most abundant in GC-MS analysis) and *α*-muurolene (the most active in molecular docking analysis) with the mosquito juvenile hormone-binding protein (mJHBP) using the Desmond 2020.1 from Schrödinger, LLC. The reproducibility was completed using three replicates for each MD run. The OPLS-2005 force field and explicit solvent model with the SPC water molecules were used in this system. To neutralize and simulate the physiological conditions, the charge Na+ ions and 0.15 M NaCl were added, respectively. The system was equilibrated using constant-temperature and constant-volume ensemble (NVT) for 100 ps. This was followed by a short run equilibration and minimization using constant-temperature and constant-pressure ensemble (NPT) for 12 ps and was set up using the Nose-Hoover chain coupling scheme with temperature 27°C, the relaxation time of 1.0 ps, pressure 1 bar, and a time of 2 fs. The Martyna-Tuckerman-Klein chain coupling scheme barostat method was used for pressure control using a relaxation time of 2 ps. The particle mesh Ewald method was used for calculating long-range electrostatic interactions with a radius of 9 Å for the coulomb interactions. Bonded forces were calculated using a RESPA integrator with a time step of 2 fs for each trajectory. The root mean square deviation (RMSD), radius of gyration (Rg), root mean square fluctuation (RMSF), and solvent accessible surface area (SAS area) were calculated to monitor the stability of the MD simulation.

### 2.9. Statistical Analysis

In the analysis of the antioxidant profile of essential oil from *S. molle*, the IC_50_ values were estimated by linear regression statistics. A Spearman's Rho coefficient was calculated to establish a correlation between the evaluated concentrations and the antioxidant response. *P* values less than 0.01 were considered statistically significant. GraphPad Prism program version 6.0 (La Jolla, CA, USA) was used to carry out the statistical analysis.

## 3. Results and Discussion

### 3.1. Chemical Profile of the Essential Oil of *S. molle*

**Figure 1:**
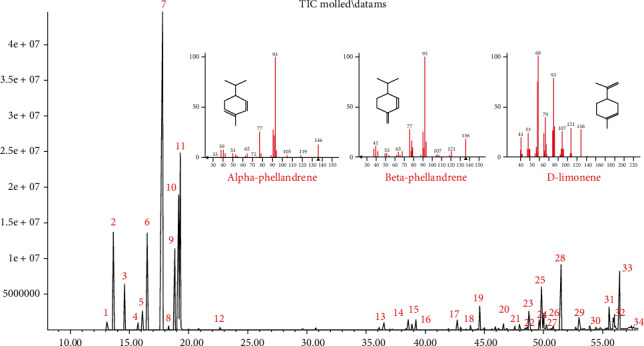
Chromatographic profile of the essential oil from *S. molle* leaves by GC-MS.

The obtained EO of fresh leaves showed a light-yellow color, a density of 0.873 ± 0.02 g/mL at 20°C, and an extraction yield of 0.73% during 2 hours of distillation. The profiles of volatile constituents of *S. molle* essential oil were analyzed by GC-MS and are presented in Figure 1 and Table 1. Regarding the chemical profile, the EO showed 34 compounds (Figure 1), two of which are of unknown structures (compounds 27 and 29), which accounted for 99.7% of the total composition. Those unknown chemical structures were classified as oxygenated sesquiterpenes with the following formula C_15_H_26_O and a molecular weight of 222.37 g/mol. The analysis identified alpha-phellandrene as the main volatile chemical with 32.68%, followed by D-limonene (12.59%) and beta-phellandrene (12.24%). According to Figure 1, the retention time at 17.80 min corresponded to the major component in the EO (compound 7).

In the phytochemical analysis, our results differed from those of other investigations and could be explained by different types of factor such as type of extraction, temperature conditions, storage conditions, edaphic conditions, plant part extracted, ecosystem, environmental, harvesting time, and the presence of environmental pollution [29]. In this study, the most representative molecule was alpha-phellandrene with 32.68%. However, in recent studies, as reported by Morales-Rabanales et al., *β*-phellandrene (15.7%) and *α*-phellandrene (12.1%) were the main components of the EO from *S. molle* leaves cultivated in Tepetitla de Lardizábal, Mexico [30]. In an analysis of *S. molle* EO obtained of dried leaves from Seropédica, Rio de Janeiro, Brazil, epi-*α*-cadinol (22.85%) was the major component [31]. Additionally, in *S. molle* from Caxias do Sul, Brazil, alpha-pinene (60.04 ± 0.07%) was the most abundant constituent [32]. In Naviraí-MS, Brazil, the major component was epi-*α*-cadinol with 21.0 ± 1.1%, followed by myrcene (16.7 ± 1.1) and sabinene (14.5 ± 0.8) [33]. *S. molle* from North Cyprus showed values of alpha-phellandrene, limonene, and beta-phellandrene equivalent to 31.5%, 10.1%, and 9.9%, respectively [34]. On the other hand, *S. molle* from Sonora, Mexico, revealed 60.8% of D-limonene (17.8%), o-cymene (16.4%), *β*-phellandrene (12.6%), *δ*-cadinene (7.5%), and caryophyllene (6.5%) [35]. However, in Jordan and Turkey, alpha-phellandrene was the most abundant constituent with 48.2% and 29.0%, respectively.

### 3.2. Antioxidant Profile of *S. molle* Essential Oil

*S. molle* EO exhibited a low antioxidant activity, as is shown in Table 2. On the other hand, there was a significant difference between the different methods used in the antioxidant activity (*P* = 0.0004). Other reports have shown different values; according to Eryigit et al., EO showed a TEAC (ABTS) = 4.7 ± 1.2 mM Trolox of *S. molle* grown in Turkey [36]. EO from Portugal reported an inhibition value of 4.8% at 16 mg/mL and was obtained by hydrodistillation. Although alpha-phellandrene, myrcene, limonene, and beta-phellandrene were the main components in this EO, its low antioxidant activity could be associated with the low ability of monoterpene hydrocarbons for DPPH scavenging activity. However, in other methods, such as beta-carotene bleaching, the antioxidant activity was high and might be justified by the presence of the monoterpenes *α* and *β*-phellandrene, *α*-pinene, sabinene, limonene, *β*-myrcene, and others [10]. In this study, the IC_50_ against DPPH was 41.84 *μ*g/mL, which was higher compared to the findings of the EO from southeast Portugal [10]. In another study, *S. molle* from Egypt showed an IC_50_ at DPPH of 172.45 *μ*g/mL and a high percentage of oxygenated sesquiterpene (17.73%) [37]. According to Table 2, the best effect was found in the FRAP assay, followed by ABTS and DPPH. It is known that DPPH and ABTS methods are based on electron and H atom transfer reaction, while the FRAP method is based on electron transfer reaction. Thus, the volatile phytochemicals from *S. molle* EO could be acted by electron transfer to reduce the free radicals.

### 3.3. Molecular Docking of the Essential Oil from *S. molle* in the Presence of the Mosquito Juvenile Hormone-Binding Protein (mJHBP) from *Aedes aegypti*

The virtual screening of the 32 chemical components, additionally JH3 and the pyriproxyfen (synthetic insecticide), was carried out in order to understand the interaction profile of various volatile compounds present in *S. molle* EO leaves with mJHBP from *A. aegypti*. Out of 32 specific compounds found abundantly in chromatography, gamma-cadinene and alpha-muurolene displayed a lower binding energy (Δ*G*) of -9.7 kcal/mol and predicted inhibitory concentration (Ki) of 0.134 *μ*M (Table 3). Although the major component (alpha-phellandrene) had a high binding free energy (Δ*G* = −7 kcal/mol), compared to alpha-muurolene, the total components might be synergizing the insecticidal effect.

**Figure 2:**
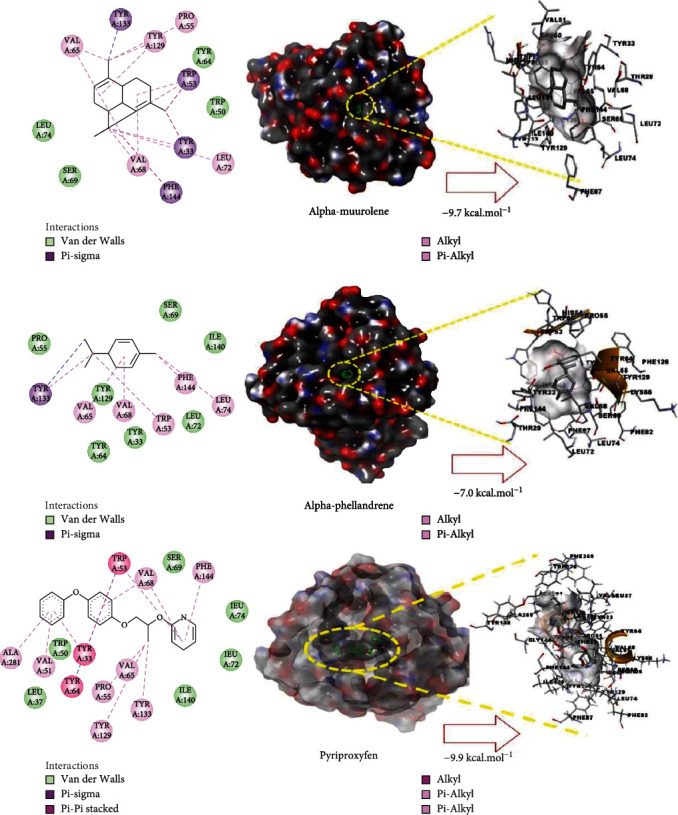
Molecular studies of the interaction of volatile constituents of *S. molle* essential oil (alpha-muurolene, alpha-phellandrene) and synthetic insecticide (pyriproxyfen) with mosquito juvenile hormone-binding protein (PDB ID: 5V13): surface view (right) and 2D (left) interactions.

The principal residues of mJHBP, TYR133, TRP53, TYR33, and PHE144 were involved in Pi-Sigma bond formation with alpha-muurolene (Figure 2). We observed that TYR133 was the main residue of mJHBP in which the molecules of the EO established a Pi-sigma, Van der Walls, and Pi-alkyl bonds (Supplementary Materials S1–S32). Interestingly, elixene, germacrene D, beta-cadinene, gamma-muurolene, germacrene-D, humulene, and gamma-eludesmol exhibited activity and binding energies close to those of the known insecticide pyriproxyfen and the cocrystallized ligand JH3. Alpha-phellandrene, beta-caryophyllene, beta-elemene, alpha-pinene, beta-phellandrene, beta-pinene, elemol, etc. have exhibited significant binding and inhibition of the mosquito juvenile hormone-binding protein. Therefore, from the docking study, it can be predicted that the molecules from this essential oil have great potential as inhibitors of the mosquito juvenile hormone-binding protein. The interactions of JH3 in this study are according to the results of Ramos et al., which the epoxy group forms a conventional hydrogen bond with the phenolic hydroxyl of Tyr-129, and other interactions showed on the isoprenoid chain were Val65,Val68, Pro55, Phe144, Tyr64, Tyr33, Ala281, Trp53, and Phe269 [18]. Furthermore, the low binding free energy (Δ*G* > −9 kcal/mol) of some volatile components was evidenced in those interactions with the Tyr133, Trp53, Tyr33, and Phe144 residues as shown in Supplementary Materials Figures S1–S32. These could infer that the insecticidal activity is related to these residues mentioned above.

### 3.4. Molecular Dynamics of the Phytoconstituents of the Essential Oil from *S. molle*

**Figure 3:**
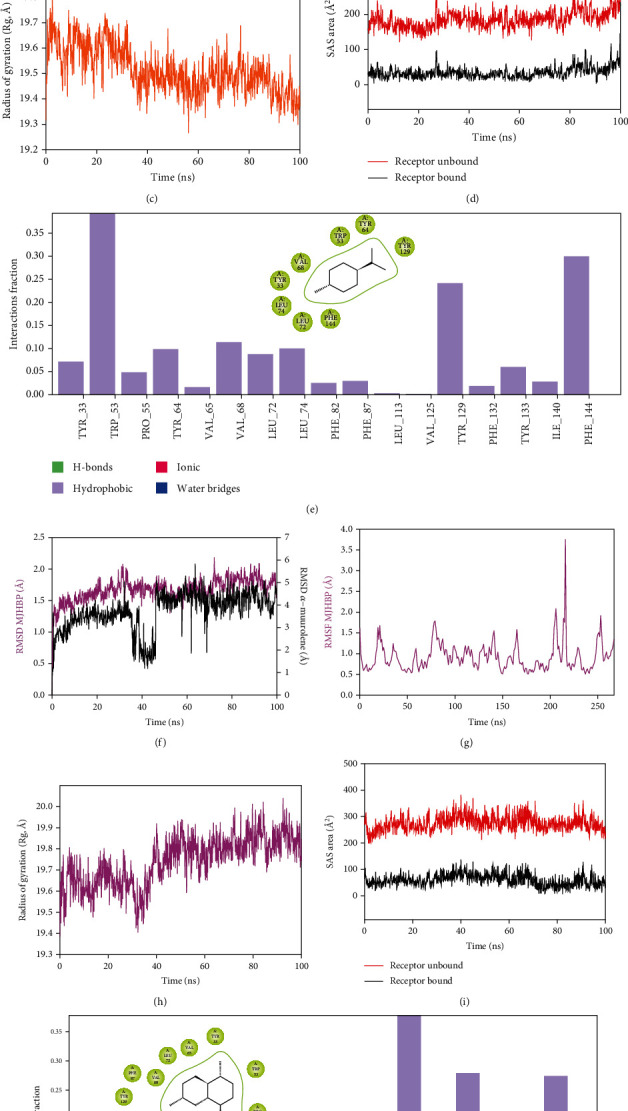
Analysis of MD simulation trajectories for 100 ns. RMSD plots displaying the molecular vibrations of C*α* backbone of (a) mJHBP+*α*-phellandrene and (f) mJHBP+*α*-muurolene. RMSF plots showing the fluctuations of respective amino acids throughout the simulation time 100 ns for (b) mJHBP+*α*-phellandrene and (g) mJHBP+*α*-muurolene. Radius of gyration plots for the deduction of compactness of protein (c) mJHBP+*α*-phellandrene and (h) mJHBP+*α*-muurolene. Solvent accessible surface area (SAS area) displaying the ligand-bound and ligand-unbound area at the binding pocket (d) mJHBP+*α*-phellandrene and (i) mJHBP+*α*-muurolene. Interaction fractions displaying the predominant hydrophobic interactions of the binding cavity residues of mJHBP with (e) *α*-phellandrene and (j) *α*-muurolene.

Molecular dynamics and simulation (MD) studies were carried out in order to determine the stability and convergence of mJHBP+*α*-phellandrene and mJHBP+*α*-muurolene complexes. Each simulation of 100 ns displayed stable conformation comparing the root mean square deviation (RMSD) values. The C*α* backbone of mJHBP bound to *α*-phellandrene exhibited a deviation of 0.5 Å (Figure 3(a)), while mJHBP bound to *α*-muurolene exhibited a deviation of 1.0 Å (Figure 3(f)). RMSD plots are within the acceptable range signifying the stability of mJHBP in the ligand-bound state before and after simulation, and it can also be suggested that ligand *α*-phellandrene and *α*-muurolene-bound mJHBP are quite stable in complex due to the higher affinity of the ligand. The plots for root mean square fluctuations (mMSF) displayed a significant spike in fluctuation (3.5 Å) at amino acid residue 220 in mJHBP, while the rest of the residues fluctuated less during the entire 100 ns simulation (Figures 3(b) and 3(g)) indicating the stable amino acid conformations during the simulation time. Therefore, from the RMSF plots, it can be suggested that the structures of mJHBP were stable during simulation in *α*-phellandrene- and *α*-muurolene-bound conformations. The radius of gyration is the measure of compactness of the protein. In this study, the mJHBP C*α* backbone displayed a lowering of the radius of gyration (Rg) from 19.6 Å to 19.4 Å, and the lowering of the Rg indicates the compactness of the complex (Figure 3(c)). On the other hand, the lowering of Rg was observed for the mJHBP+*α*-muurolene complex till 35 ns, and a later increment of the peak was observed. This indicates the less stable conformation of mJHBP+*α*-muurolene as compared to mJHBP+*α*-phellandrene. However, the stable Rg peak confirmed the significant compactness of the protein in the *α*-muurolene-bound state (Figure 3(h)). The overall quality analysis from RMSD and Rg suggests that *α*-phellandrene and *α*-muurolene bound to the protein targets posthumously in the binding cavities and played a significant role in stability of the proteins. Solvent accessible surface area provides the information about the compactness of protein complex with the ligand. The lowering of SASA in the case of *α*-phellandrene and *α*-muurolene bound to mJHBP as compared to the unbound state signifies the achievement of stable converged structures due to the high compactness of both the systems (Figures 3(d) and 3(i)). The interaction plots of both *α*-phellandrene and *α*-muurolene bound to mJHBP displayed no involvement of conventional hydrogen bonds, while hydrophobic interactions played a major role in ligand stabilization at the binding cavity of mJHBP (Figures 3(e) and 3(j)).

## 4. Conclusions

The essential oil of *S. molle* leaves presented 34 volatile constituents determined by gas chromatography-mass spectrometry (GC-MS). Alpha-phellandrene was the major component, which represented 32.68% of the total composition; furthermore, beta-phellandrene and D-limonene were the following more abundant metabolites with 12.24% and 12.59%, respectively. Additionally, the antioxidant activity was evaluated in order to determine the antioxidant profile of EO using the three methods of DPPH, ABTS, and FRAP. According to the results, the EO showed better affinity and a good effect on FRAP assay and IC_50_ equivalent to 1.50 ± 0.02 mg/mL. However, this study revealed that *S. molle* EO is not a good antioxidant. Regarding the insecticidal activity in silico based on a virtual screening on mosquito juvenile hormone-binding protein, several volatile compounds were active against this target such as alpha-muurolene and gamma-cadinene, being similar to pyriproxyfen, which is a synthetic insecticide analogue of JH. The molecular dynamics carried out for alpha muurolene (the best result in molecular docking) and alpha-phellandrene (the most abundant molecule) were very stable during 100 ns of evaluation. In the future, *S. molle* EO might be used as bioinsecticide on *A aegypti*, but an in vitro and in vivo assay has to be evaluated to validate our findings.

## Figures and Tables

**Table 1:** Chemical composition of the volatile oil of *Schinus molle* leaves.

Compound name	Rt (min)	Molecular formula/molecular mass	%	LRI ^Exp^	LRI ^Ref^	Chemical structure	Chemical group
Tricyclene	13.13	C_10_H_16_ (136.23)	0.49	926	926	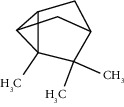	Monoterpene hydrocarbon
Alpha-pinene	13.65	C_10_H_16_ (136.23)	5.27	930	932	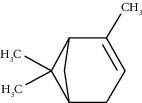	Monoterpene hydrocarbon
Camphene	14.58	C_10_H_16_ (136.23)	2.50	938	946	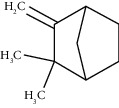	Monoterpene hydrocarbon
Sabinene	15.72	C_10_H_16_ (136.23)	0.40	973	974	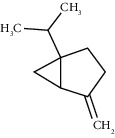	Monoterpene hydrocarbon
Beta-pinene	16.10	C_10_H_16_ (136.23)	1.11	976	975	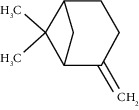	Monoterpene hydrocarbon
Beta-myrcene	16.51	C_10_H_16_ (136.23)	5.94	988	988	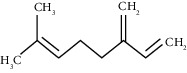	Monoterpene hydrocarbon
Alpha-phellandrene	17.80	C_10_H_16_ (136.23)	32.68	1006	1006	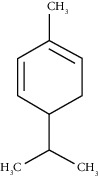	Monoterpene hydrocarbon
Alpha-terpinene	18.31	C_10_H_16_ (136.23)	0.26	1019	1020	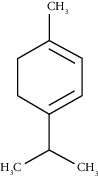	Monoterpene hydrocarbon
o-Cymene	18.82	C_10_H_14_ (136.22)	5.58	1022	1022	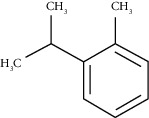	Aromatic monoterpene hydrocarbon
D-Limonene	19.16	C_10_H_16_ (136.23)	12.59	1027	1024	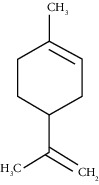	Monoterpene hydrocarbon
Beta-phellandrene	19.32	C_10_H_16_ (136.23)	12.24	1029	1025	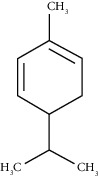	Monoterpene hydrocarbon
Terpinolene	22.65	C_10_H_16_ (136.23)	0.21	1082	1086	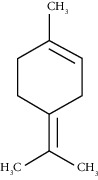	Monoterpene hydrocarbon
Bornyl acetate	36.12	C_12_H_20_O_2_ (196.29)	0.20	1277	1284	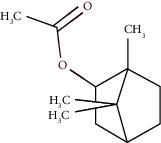	Oxygenated monoterpene
Gamma-elemene	39.20	C_15_H_24_ (204.35)	0.68	1650	1651	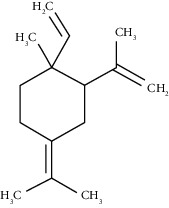	Sesquiterpene hydrocarbon
Beta-elemene	42.71	C_15_H_24_ (204.35)	0.72	1382	1389	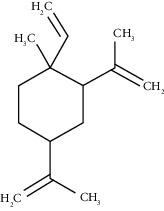	Sesquiterpene hydrocarbon
Beta-gurjunene	43.83	C_15_H_24_ (204.35)	0.30	1402	1409	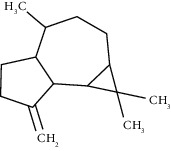	Sesquiterpene hydrocarbon
Beta-caryophyllene	44.60	C_15_H_24_ (204.35)	0.30	1416	1417	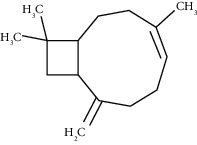	Sesquiterpene hydrocarbon
Elixene	44.99	C_15_H_24_ (204.35)	0.26	1441	1445	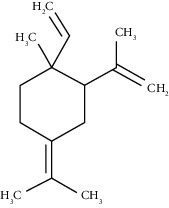	Sesquiterpene hydrocarbon
Humulene	46.62	C_15_H_24_ (204.35)	0.39	1450	1452	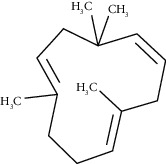	Sesquiterpene hydrocarbon
Gamma-muurolene	47.58	C_15_H_24_ (204.35)	0.21	1452	1451	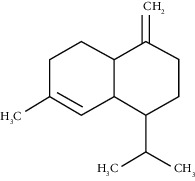	Sesquiterpene hydrocarbon
Germacrene D	47.99	C_15_H_24_ (204.35)	0.38	1471	1480	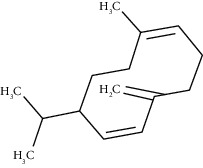	Sesquiterpene hydrocarbon
Bicyclogermacrene	48.77	C_15_H_24_ (204.35)	1.30	1492	1500	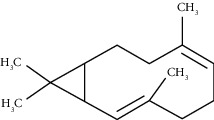	Sesquiterpene hydrocarbon
Alpha-muurolene	48.83	C_15_H_24_ (204.35)	0.60	1509	1510	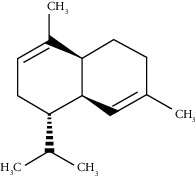	Sesquiterpene hydrocarbon
*γ*-Cadinene	49.66	C_15_H_24_ (204.35)	0.62	1514	1513	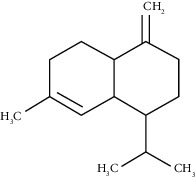	Sesquiterpene hydrocarbon
Beta-cadinene	49.87	C_15_H_24_ (204.35)	2.95	1515	1522	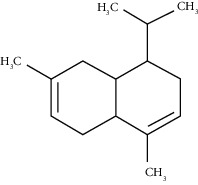	Sesquiterpene hydrocarbon
Shyobunol	50.03	C_15_H_26_O (222.37)	1.11	1541	1542	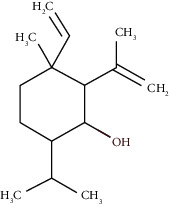	Oxygenated sesquiterpene
Unknown I	50.27	C_15_H_26_O (222.37)	0.67	1600		n.d.	Oxygenated sesquiterpene
Elemol	51.48	C_15_H_26_O (222.37)	4.53	1610	1610	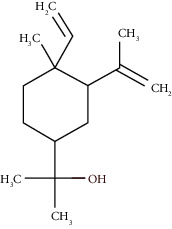	Oxygenated sesquiterpene
Unknown II	52.97	C_15_H_26_O (222.37)	1.13	1623		n.d.	Oxygenated sesquiterpene
Viridiflorol	53.91	C_15_H_26_O (222.37)	0.31	1625	1627	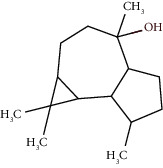	Oxygenated sesquiterpene
Gamma-eudesmol	55.59	C_15_H_26_O (222.37)	1.22	1636	1630	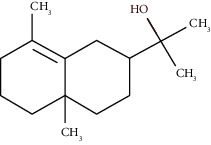	Oxygenated sesquiterpene
*τ*-Cadinol	55.93	C_15_H_26_O (222.37)	0.61	1648	1652	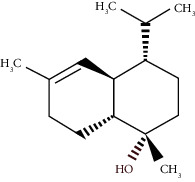	Oxygenated sesquiterpene
*τ*-Muurolol	55.99	C_15_H_26_O (222.37)	1.71	1641	1643	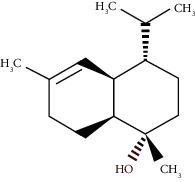	Oxygenated sesquiterpene
Delta-cadinol	56.08	C_15_H_26_O (222.37)	0.23	1655	1656	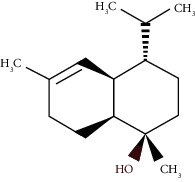	Oxygenated sesquiterpene
Aromatic monoterpene hydrocarbons			5.58%				
Monoterpene hydrocarbons			73.69%				
Oxygenated monoterpenes			0.20%				
Sesquiterpene hydrocarbons			8.71%				
Oxygenated sesquiterpenes			11.52%				
Total identified			99.7%				

Rt: retention time; n.d.: not determined; LRI ^Ref^: linear retention index obtained from the literature [25]; LRI ^Exp^: linear retention index calculated against n-alkanes C9–C24. ^a^Mean of three determinations.

**Table 2:** Antioxidant profile of the essential oil of *S. molle.*

Method	Mean ± SD
TEAC DPPH (*μ*mol TE/g)	11.42 ± 0.08
IC_50_ DPPH (mg/mL)	41.84 ± 0.31
TEAC ABTS (*μ*mol TE/g)	134.88 ± 4.37
IC_50_ ABTS (mg/mL)	2.05 ± 0.07
TEAC FRAP (*μ*mol TE/g)	65.16 ± 1.46
IC_50_ FRAP (mg/mL)	1.50 ± 0.02

**Table 3:** Ligand interaction energies and inhibitory concentrations with mosquito juvenile hormone-binding protein in the molecular docking study.

Ligand	Binding free energy (Δ*G*, kcal/mol)	Ki (*μ*M)
Alpha-phellandrene	-7	14.6
Alpha-terpinene	-6.5	58.46
Beta-caryophyllene	-8.3	1.22
Beta-myrcene	-6.5	58.46
Beta-elemene	-8.3	1.22
Bicyclogermacrene	-7.6	7.46
Camphene	-7.2	13.06
D-Limonene	-7.3	12.57
Elixene	-9	0.398
Germacrene D	-9.5	0.226
Alpha-muurolene	-9.7	0.134
Alpha-pinene	-7.5	8.19
Beta-cadinene	-9.6	0.176
Beta-gurjunene	-8.2	1.67
Beta-phellandrene	-7.5	8.19
Beta-pinene	-7.5	8.19
Elemol	-7.7	6.15
*γ*-Cadinene	-9.7	0.134
Gamma-muurolene	-9.5	0.226
Bornyl acetate	-7.8	5.56
Humulene	-9.2	0.319
o-Cymene	-7	14.6
Sabinene	-7.3	12.57
t-Cadinol	-7.8	5.56
Terpinolene	-7.5	8.19
Tricyclene	-7	14.6
Gamma-Elemene	-7.6	7.46
Shyobunol	-9.1	0.332
Gamma-eudesmol	-8.9	0.913
Delta-cadinol	-7.8	5.56
*τ*-Muurolol	-8.4	1.01
Viridiflorol	-7.7	6.15
Pyriproxyfen (synthetic insecticide)	-9.9	0.099
Juvenile hormone III (JH3)	-9.2	0.319

## Data Availability

The data used to support the findings of this study are included within the supplementary information file.

## Supplementary Materials

Supplementary Materials Figures S1–S32: molecular interaction studies of the phytochemical components from *S. molle* and mosquito juvenile hormone-binding protein (mJHBP) from *Aedes aegypti.*
