# Supplementary material for: GC-MS Profile, Antioxidant Activity, and In Silico Study of the Essential Oil from Schinus molle L. Leaves in the Presence of Mosquito Juvenile Hormone-Binding Protein (mJHBP) from Aedes aegypti
Source: Biomed Res Int. 2022 May 16;2022:5601531. doi: 10.1155/2022/5601531 (PMC9126701; doi:10.1155/2022/5601531)
Supplement: Supplementary Materials — Figures S1–S32: molecular interaction studies of the phytochemical components from S. molle and mosquito juvenile hormone-binding protein (mJHBP) from Aedes aegypti. [file 5601531.f1.docx]

**Supplementary Materials**

GC-MS Profile, Antioxidant Activity and In Silico Study of the Essential Oil from *Schinus molle* L. Leaves on Mosquito Juvenile Hormone-Binding Protein (mJHBP) from *Aedes aegypti*

Oscar Herrera-Calderon ^1,^*, Haydee Chavez ^2^, Edwin Carlos Enciso-Roca ^3^, Pablo Williams Común-Ventura ^3^, Renan Dilton Hañari-Quispe ^4^, Linder Figueroa-Salvador ^5^, Eddie Loyola Gonzales ^6^, Josefa Bertha Pari-Olarte ^2^, Nada H. Aljarba ^7^, Saad Alkahtani ^8^, Gaber El-Saber Batiha ^9^

^1^ Department of Pharmacology, Bromatology and Toxicology, Faculty of Pharmacy and Biochemistry, Universidad Nacional Mayor de San Marcos, Lima, Peru

^2^ Department of Pharmaceutical Chemistry, Faculty of Pharmacy and Biochemistry, Universidad Nacional San Luis Gonzaga, Ica 11001, Peru

^3^ Department of Human Medicine, Faculty of Health Sciences, Universidad Nacional de San Cristobal de Huamanga, Portal Independencia 57, Ayacucho 05003, Peru

^4^ Clinical Pathology Laboratory, Faculty of Veterinary Medicine and Zootechnics, Universidad Nacional del Altiplano, Av Floral 1153, Puno 21001, Peru

^5^ School of Medicine, Faculty of Health Sciences, Universidad Peruana de Ciencias Aplicadas, Prolongación Primavera 2390, Lima 15023, Peru

^6^  Department of Pharmaceutical Science, Faculty of Pharmacy and Biochemistry, Universidad Nacional San Luis Gonzaga, Ica 11001, Peru

^7^ Department of Biology, College of Science, Princess Nourah Bint Abdulrahman University, P. O. Box 84428,

Riyadh 11671, Saudi Arabia

^8^  Department of Zoology, College of Science, King Saud University, P. O. Box 2455, Riyadh 11451, Saudi Arabia

^9^ Department of Pharmacology and Therapeutics, Faculty of Veterinary Medicine, Damanhour University, Damanhour 22511, AlBeheira, Egypt

***** Correspondence: oherreraca@unmsm.edu.pe; Tel.: +51 956-550-510

***** Correspondence: oherreraca@unmsm.edu.pe; Tel.: +51-956-550-510

**
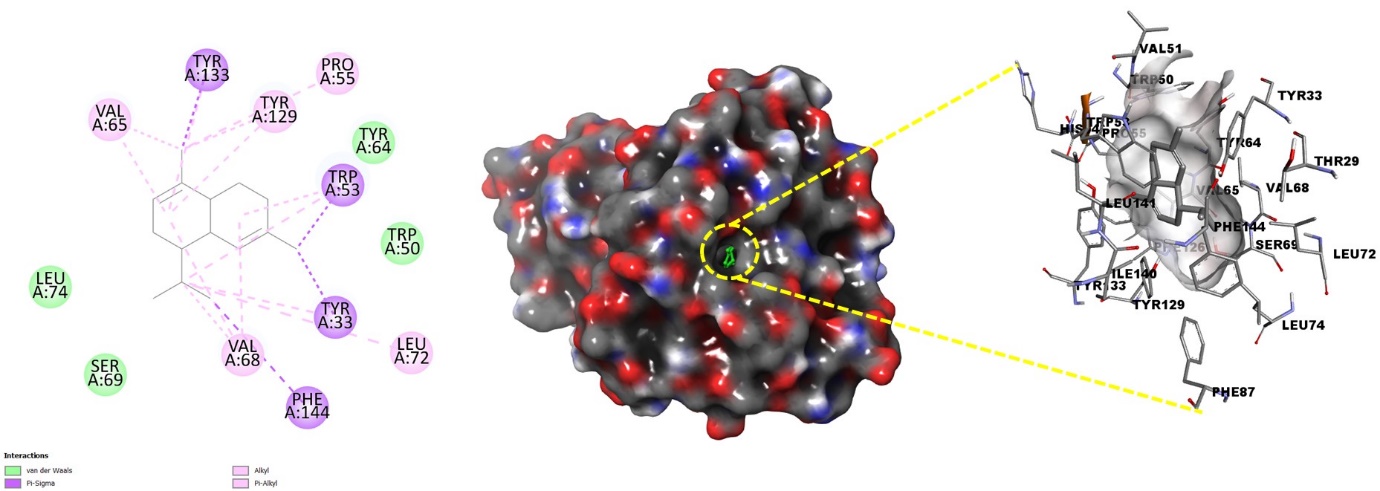
**

**Figure S1. Molecular interaction studies of alpha-muurolene with mJHBP (PDB ID: 5V13), surface view (Right panel), and 2D (Left panel) interactions.**

**
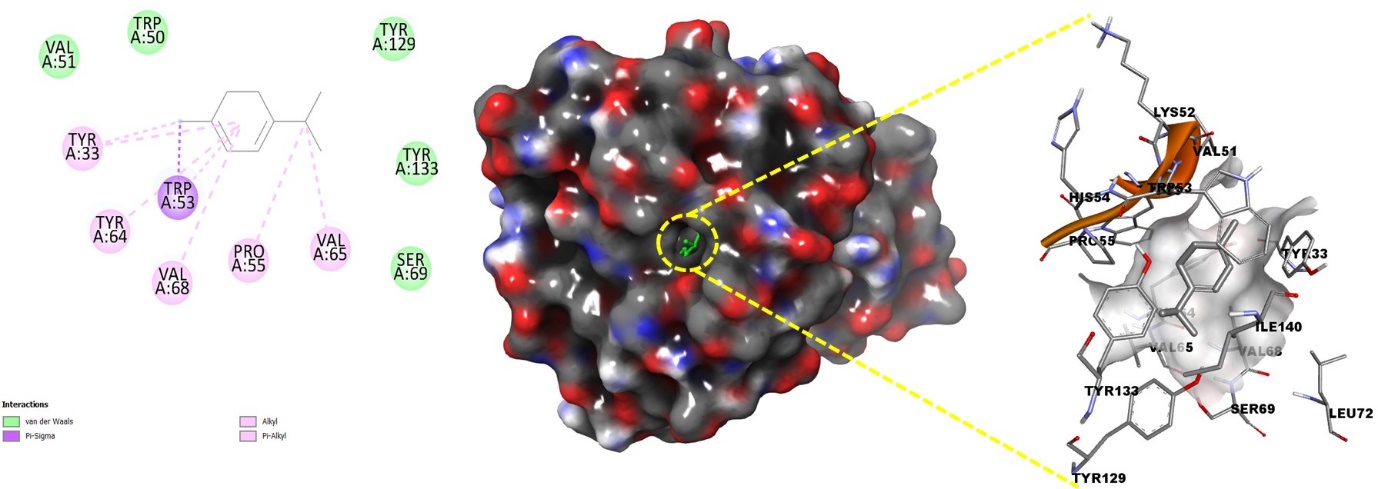
**

**Figure S2. Molecular interaction studies of alpha-terpinene with mJHBP (PDB ID: 5V13), surface view (Right panel), and 2D (Left panel) interactions.**

**
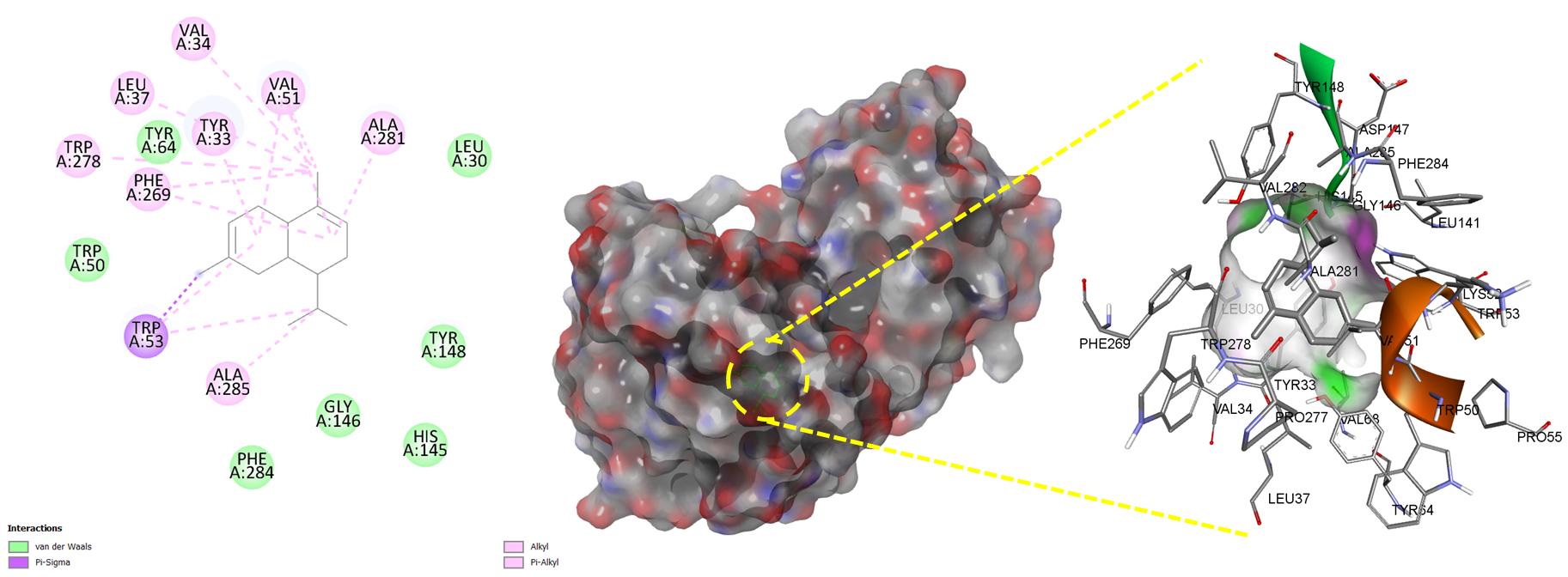
**

**Figure S3. Molecular interaction studies of beta-cadinene with mJHBP (PDB ID: 5V13), surface view (Right panel), and 2D (Left panel) interactions.**

**
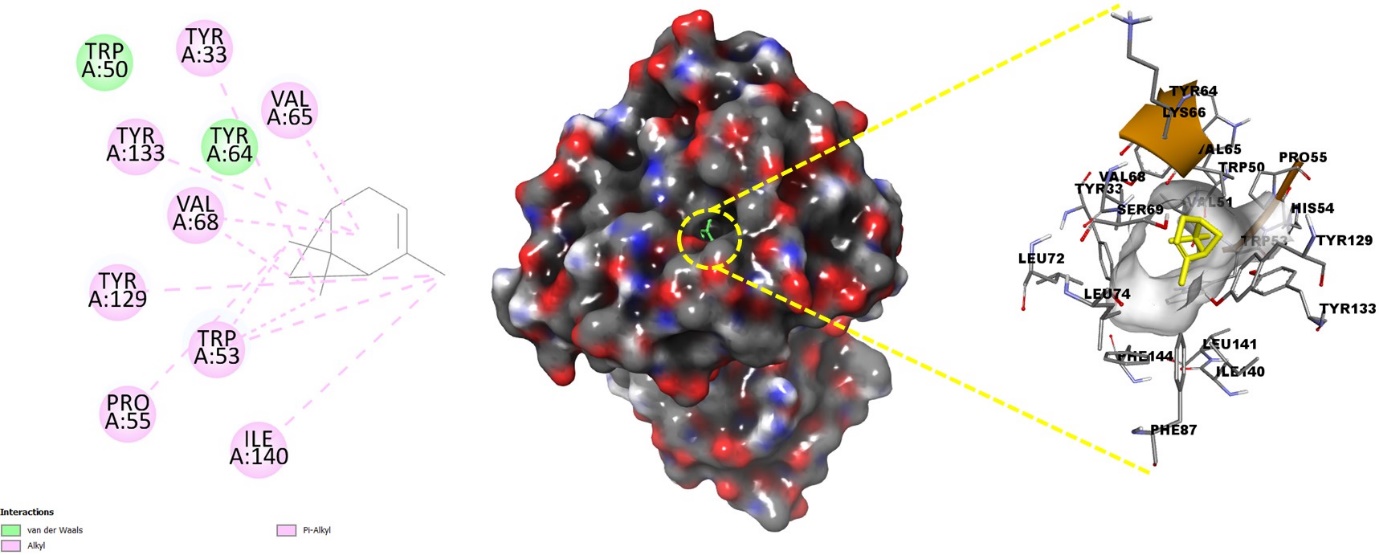
**

**Figure S4. Molecular interaction studies of alpha-pinene with mJHBP (PDB ID: 5V13), surface view (Right panel), and 2D (Left panel) interactions.**

**
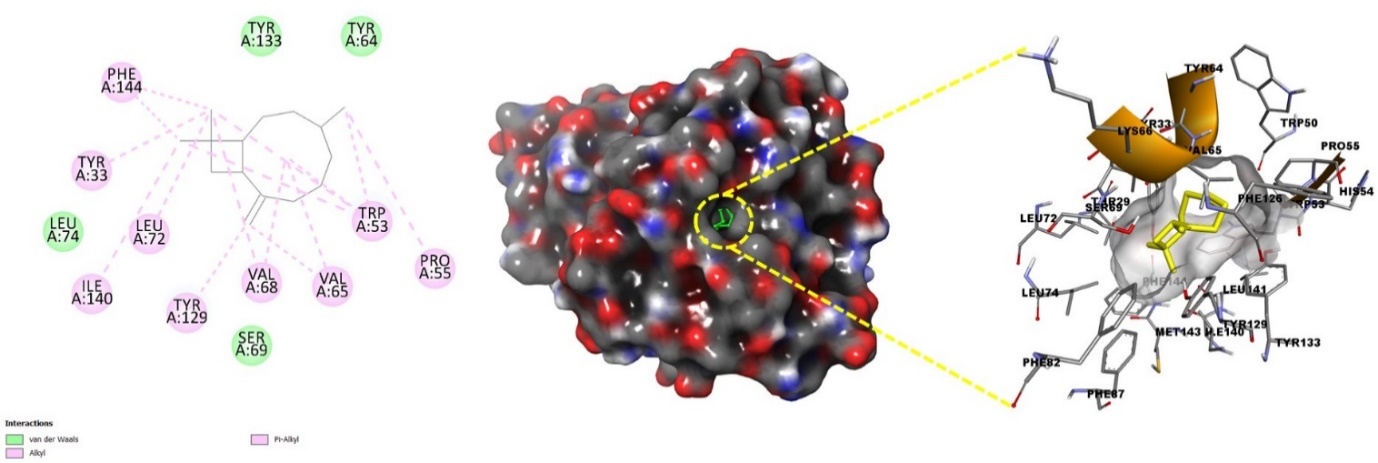
**

**Figure S5. Molecular interaction studies of beta-caryophyllene with mJHBP (PDB ID: 5V13), surface view (Right panel), and 2D (Left panel) interactions.**

**
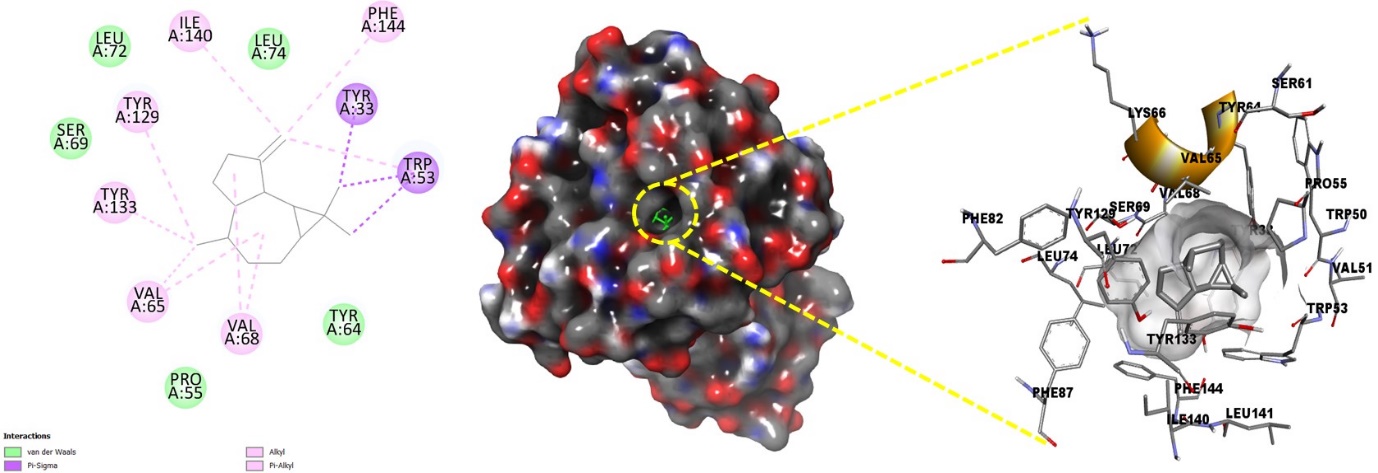
**

**Figure S6. Molecular interaction studies of beta-gurjunene with mJHBP (PDB ID: 5V13), surface view (Right panel), and 2D (Left panel) interactions.**


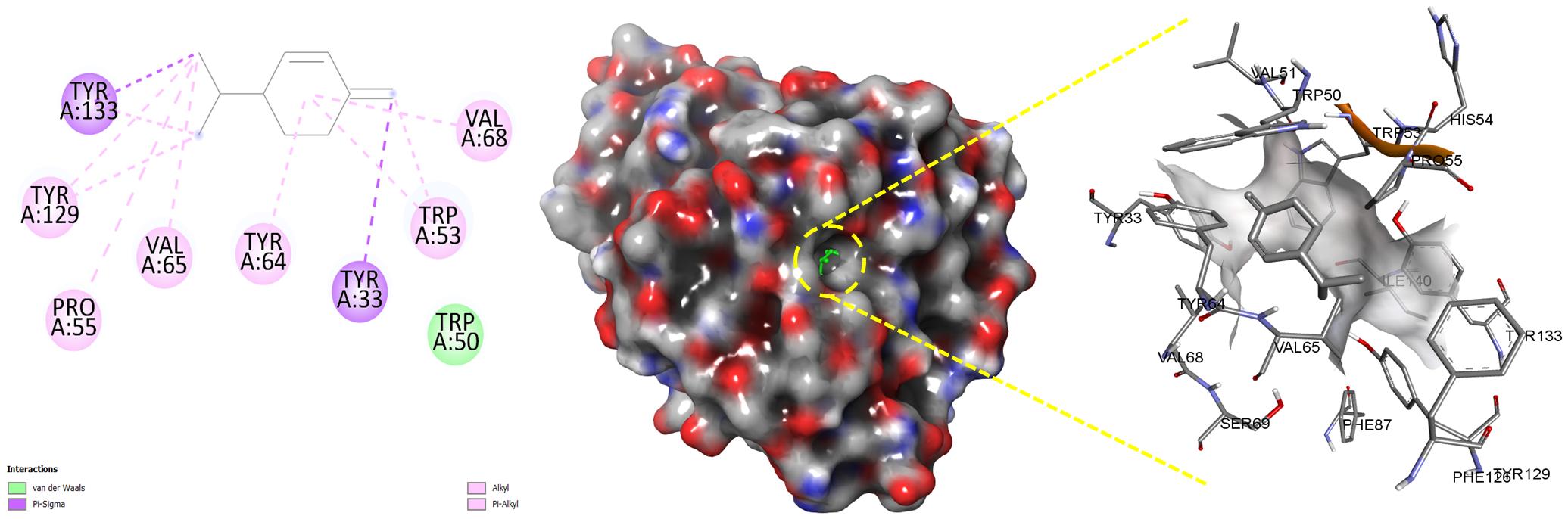


**Figure S7. Molecular interaction studies of beta-phellandrene with mJHBP (PDB ID: 5V13), surface view (Right panel), and 2D (Left panel) interactions.**

**
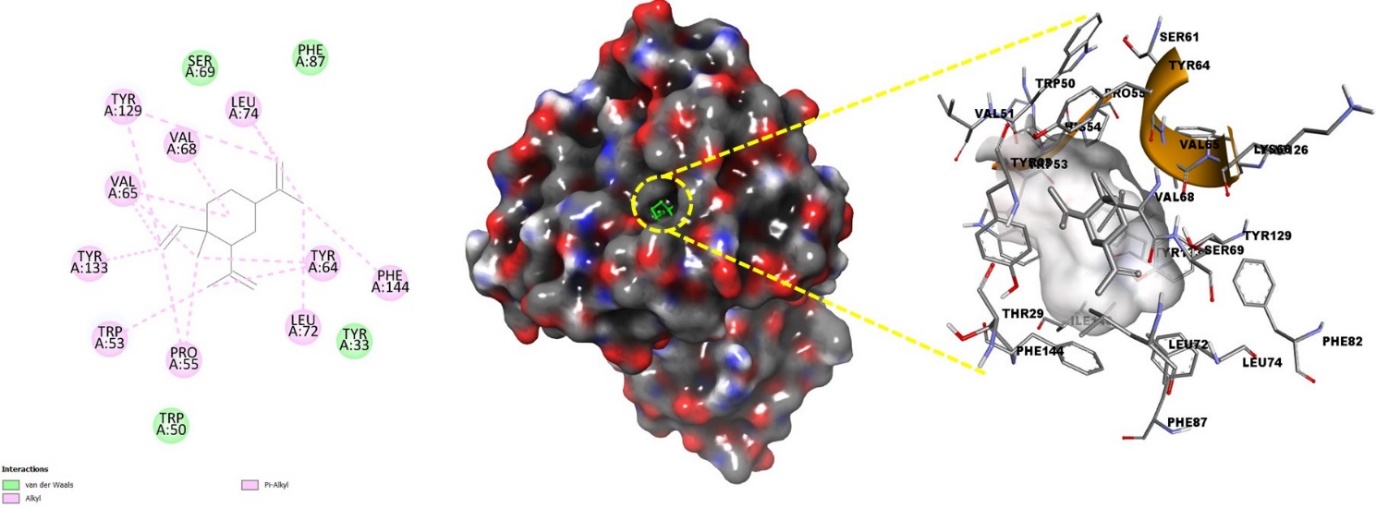
**

**Figure S8. Molecular interaction studies of beta-elemene with mJHBP (PDB ID: 5V13), surface view (Right panel), and 2D (Left panel) interactions.**

**
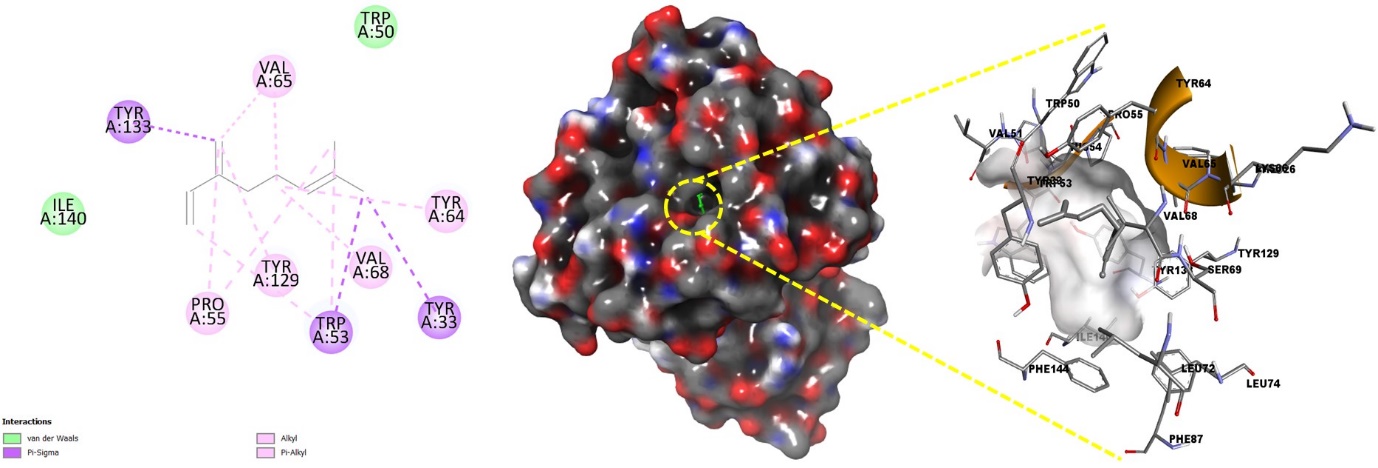
**

**Figure S9. Molecular interaction studies of beta-myrcene with mJHBP (PDB ID: 5V13), surface view (Right panel), and 2D (Left panel) interactions.**

**
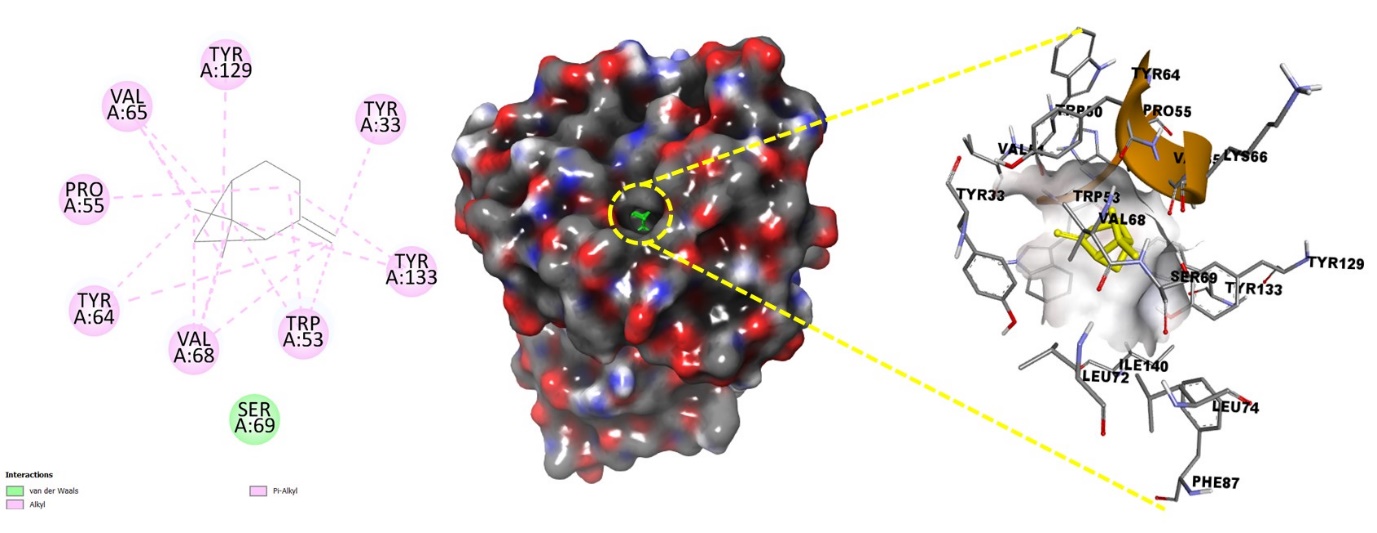
**

**Figure S10. Molecular interaction studies of beta-pinene with mJHBP (PDB ID: 5V13), surface view (Right panel), and 2D (Left panel) interactions.**

**
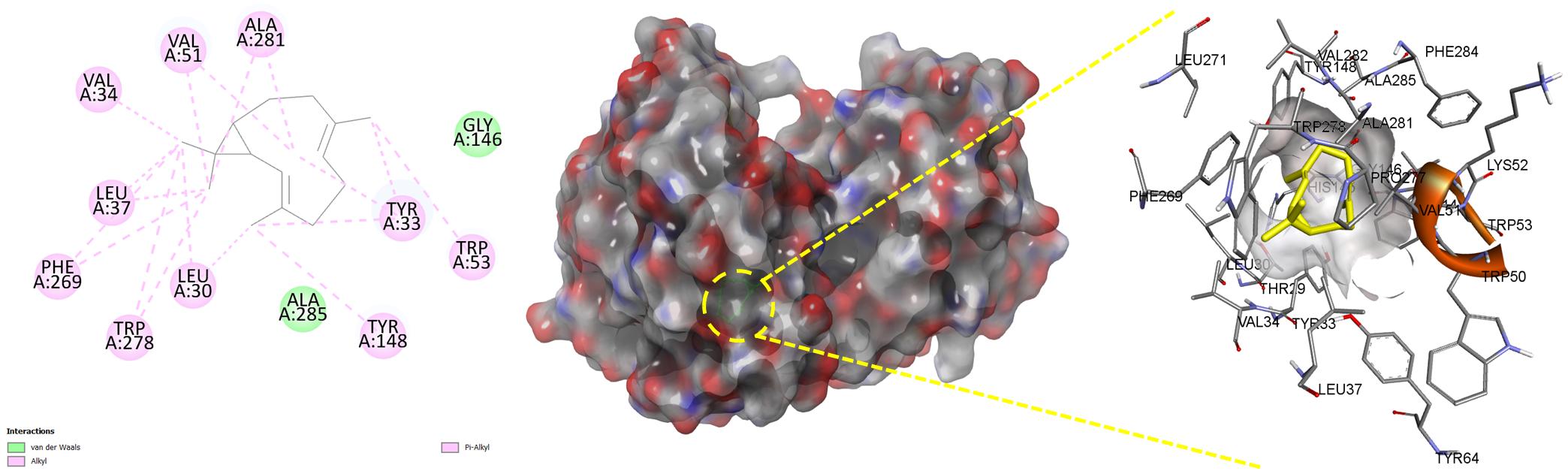
**

**Figure S11. Molecular interaction studies of bicyclogermacrene with mJHBP (PDB ID: 5V13), surface view (Right panel), and 2D (Left panel) interactions.**

**
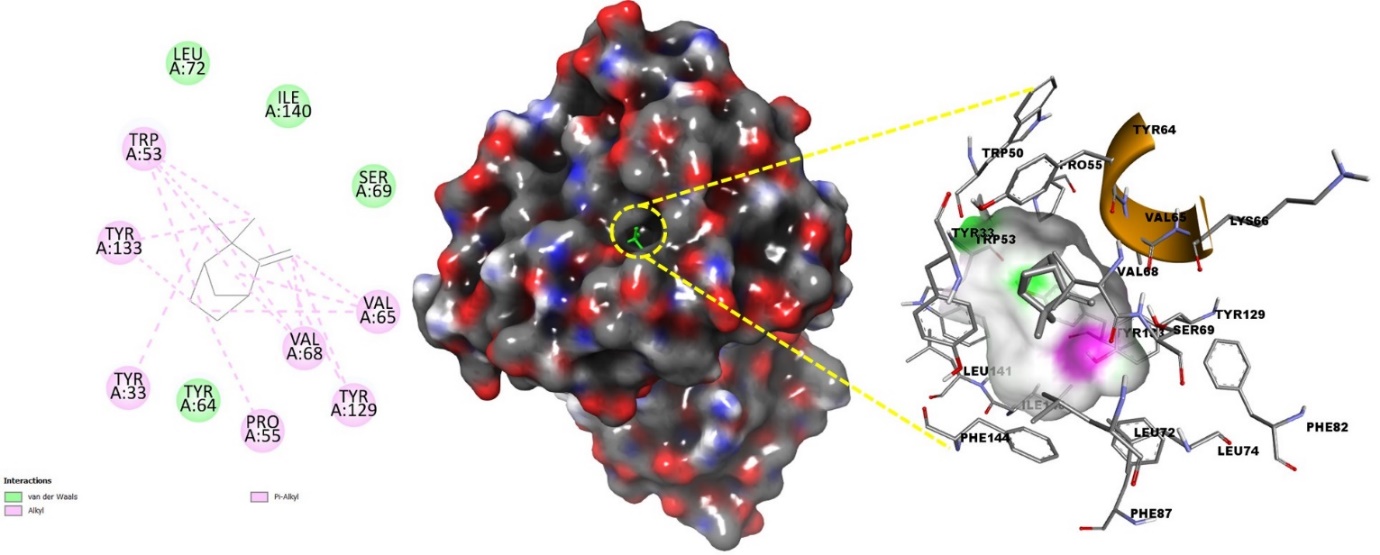
**

**Figure S12. Molecular interaction studies of camphene with mJHBP (PDB ID: 5V13), surface view (Right panel), and 2D (Left panel) interactions.**

**
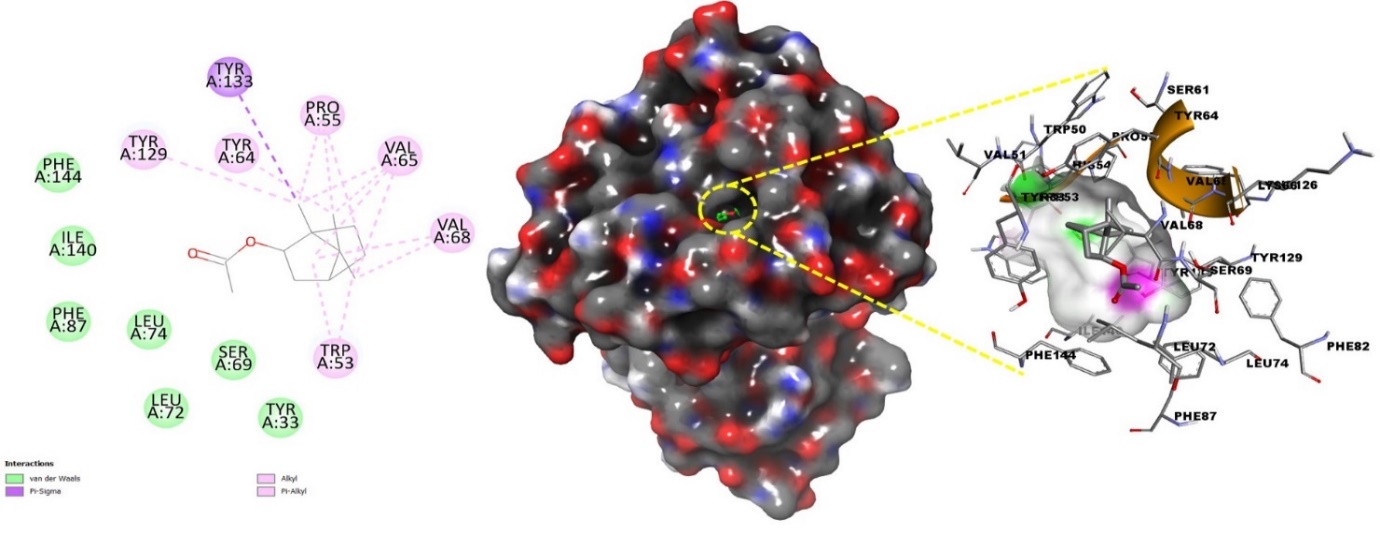
**

**Figure S13. Molecular interaction studies of borneol acetate with mJHBP (PDB ID: 5V13), surface view (Right panel), and 2D (Left panel) interactions.**

**
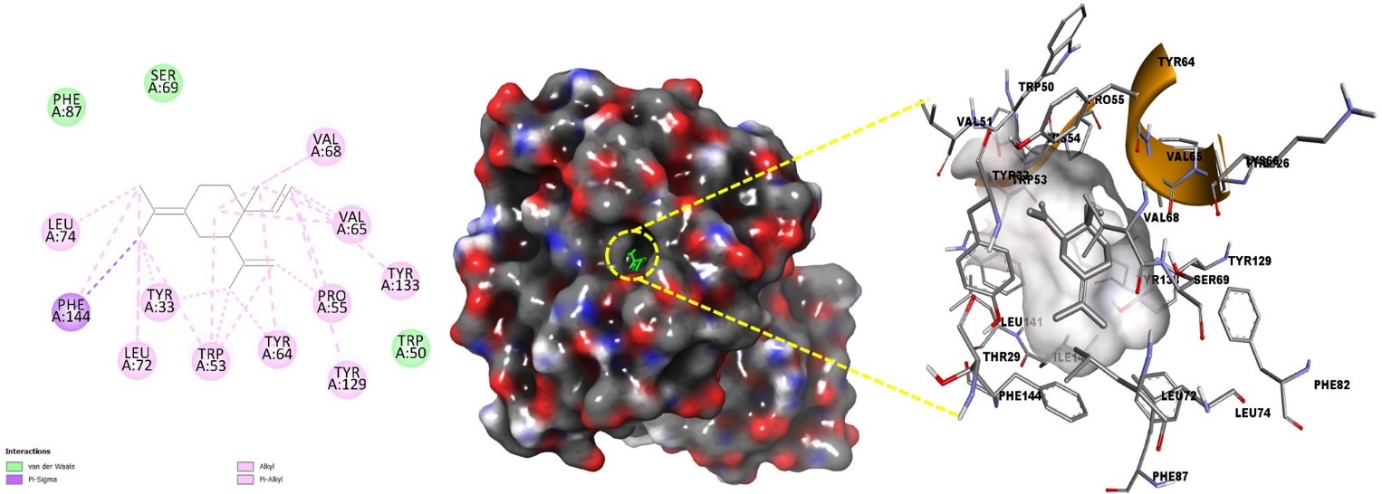
**

**Figure S14. Molecular interaction studies of cadinol with mJHBP (PDB ID: 5V13), surface view (Right panel), and 2D (Left panel) interactions.**

**
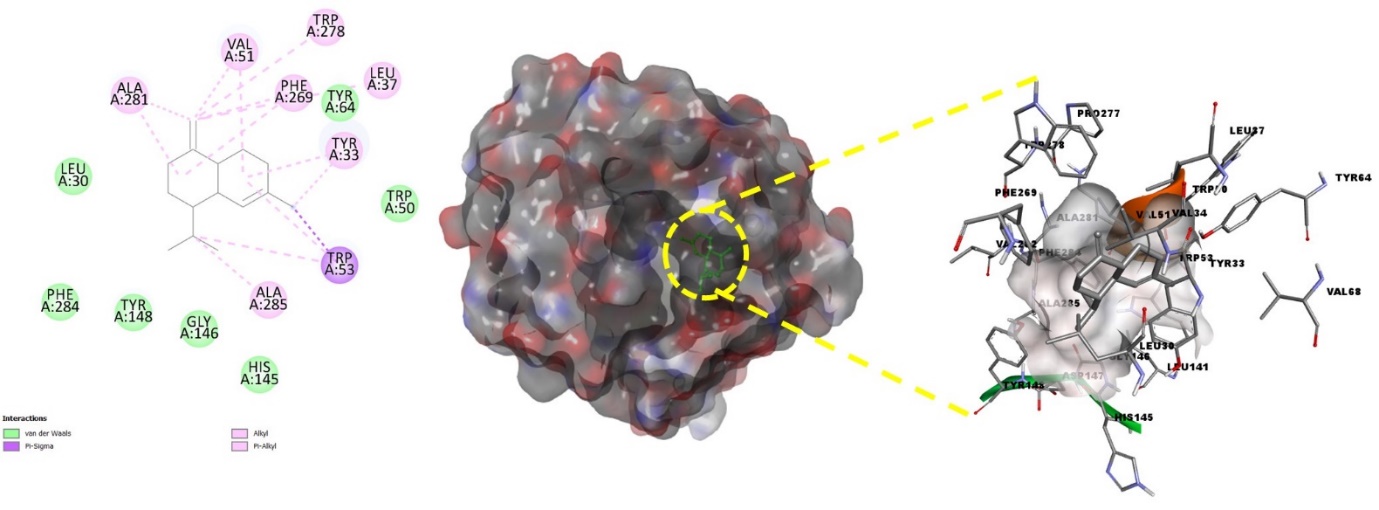
**

**Figure S15. Molecular interaction studies of gamma-muurolene with mJHBP (PDB ID: 5V13), surface view (Right panel), and 2D (Left panel) interactions.**

**
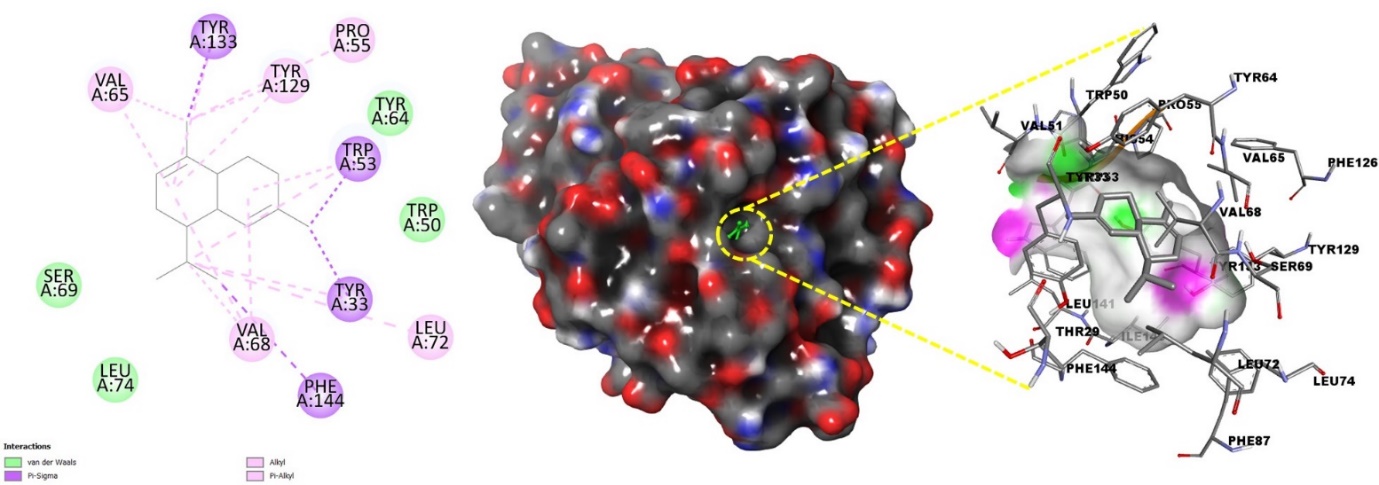
**

**Figure S16. Molecular interaction studies of gamma-cadinene with mJHBP (PDB ID: 5V13), surface view (Right panel), and 2D (Left panel) interactions.** **Tyr133, Trp53, Tyr33, Phe144.**

**
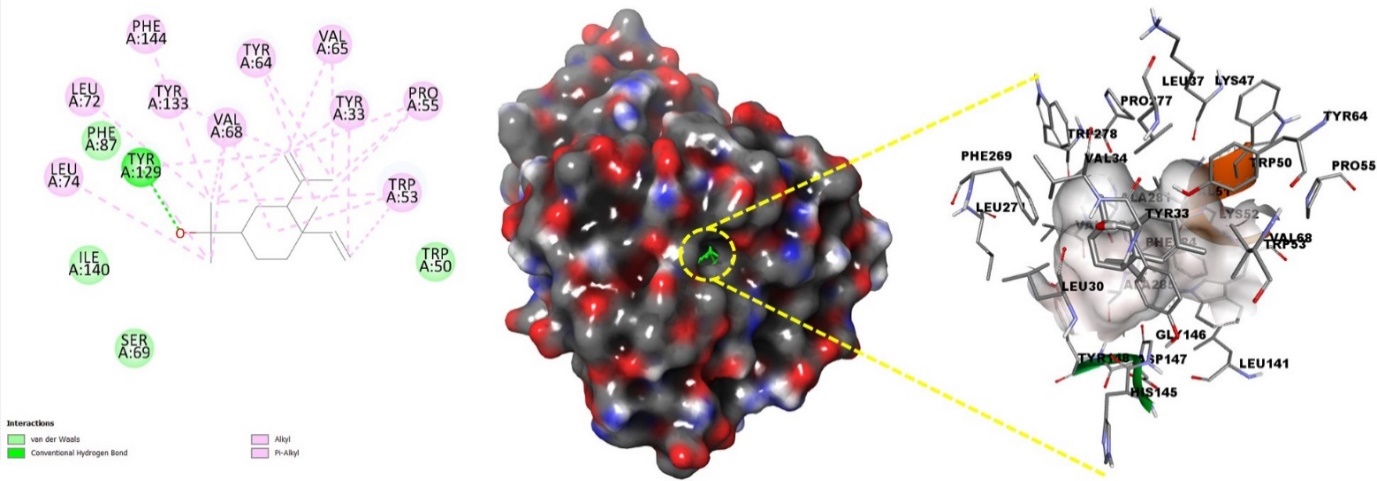
**

**Figure S17. Molecular interaction studies of elemol with mJHBP (PDB ID: 5V13), surface view (Right panel), and 2D (Left panel) interactions.**

**
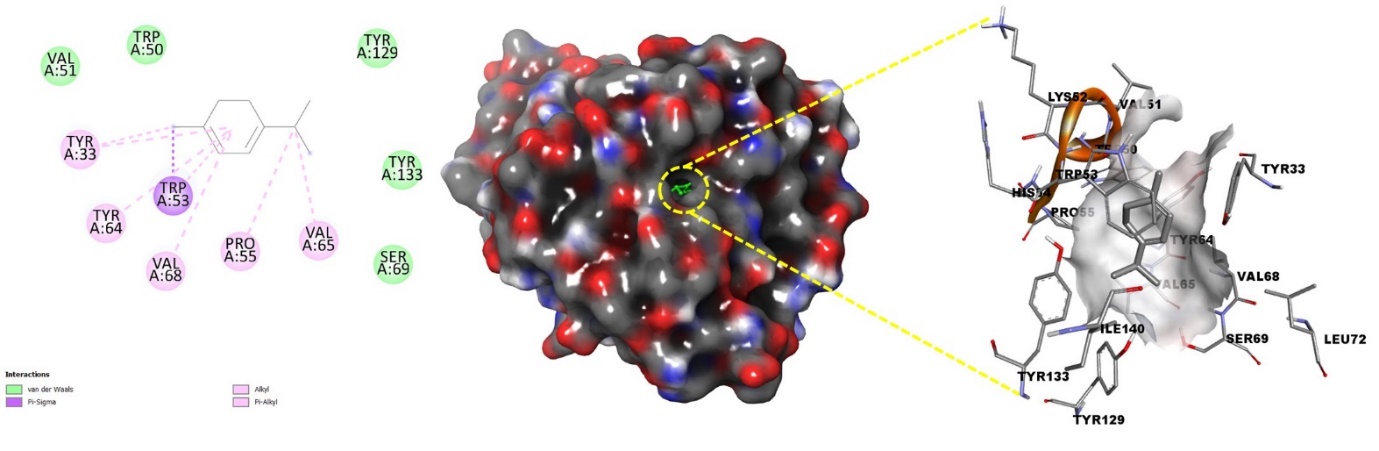
**

**Figure S18. Molecular interaction studies of D-limonene with mJHBP (PDB ID: 5V13), surface view (Right panel), and 2D (Left panel) interactions.**

**
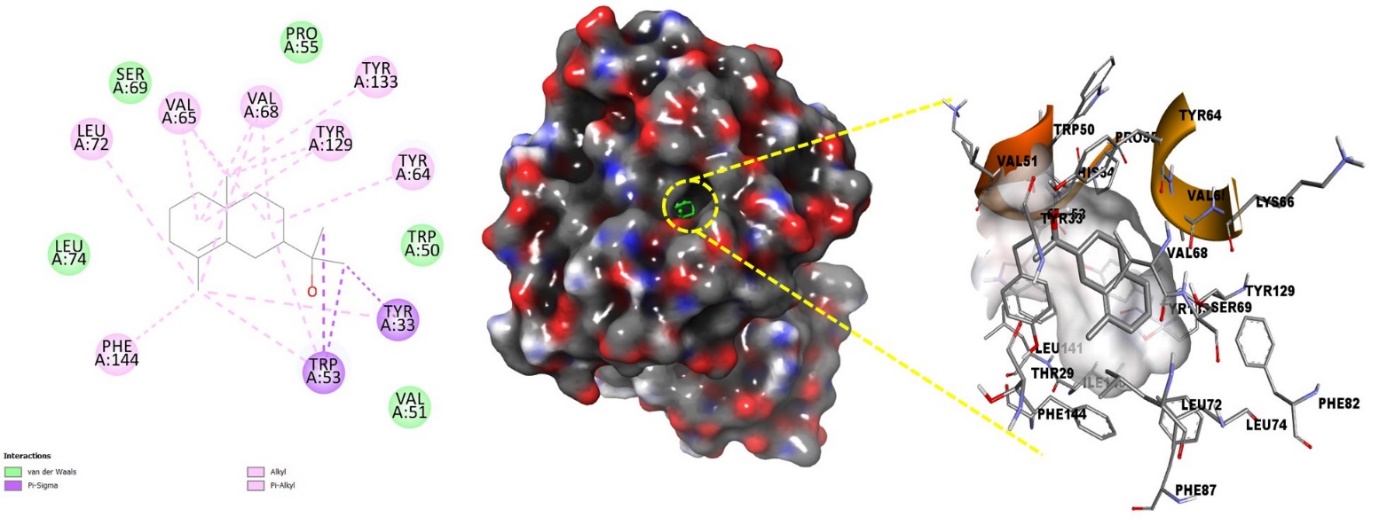
**

**Figure S19. Molecular interaction studies of eudesmol with mJHBP (PDB ID: 5V13), surface view (Right panel), and 2D (Left panel) interactions.**

**
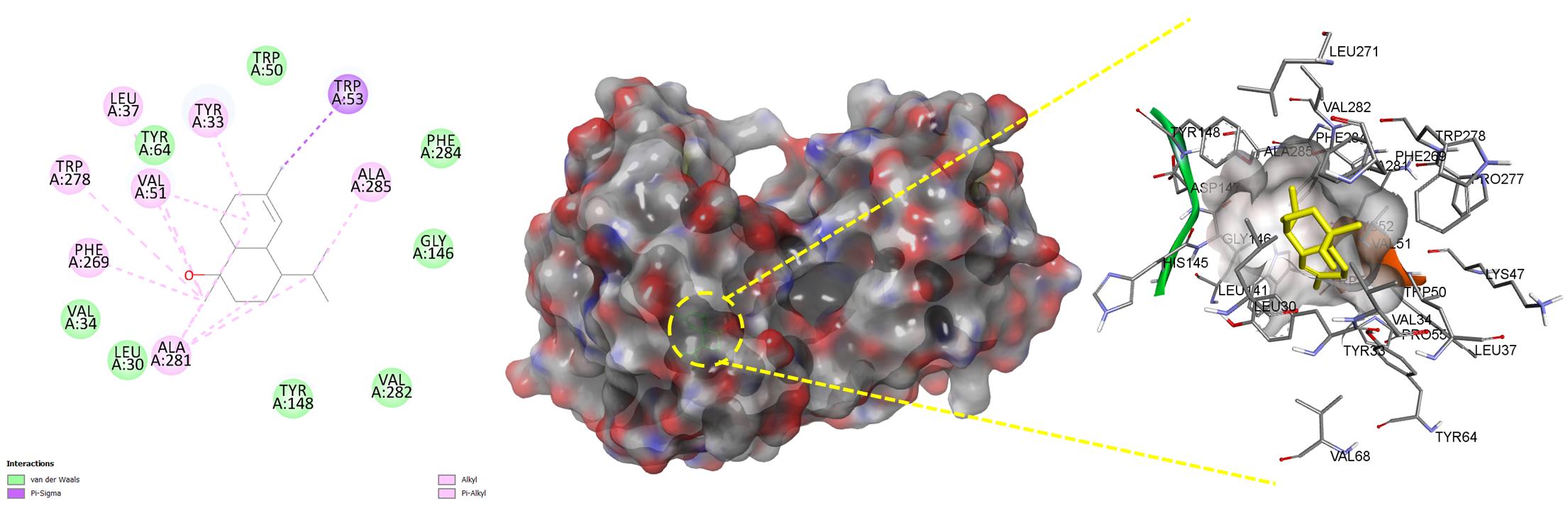
**

**Figure S20. Molecular interaction studies of t-cadinol with mJHBP (PDB ID: 5V13), surface view (Right panel), and 2D (Left panel) interactions.**

**
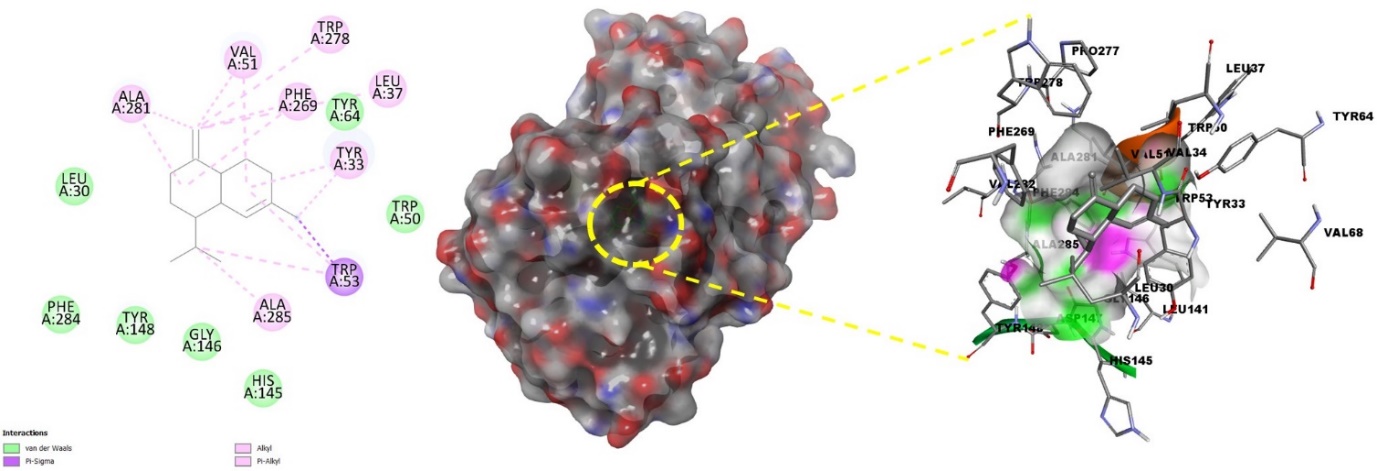
**

**Figure S21. Molecular interaction studies of germacrene-D with mJHBP (PDB ID: 5V13), surface view (Right panel), and 2D (Left panel) interactions.**

**
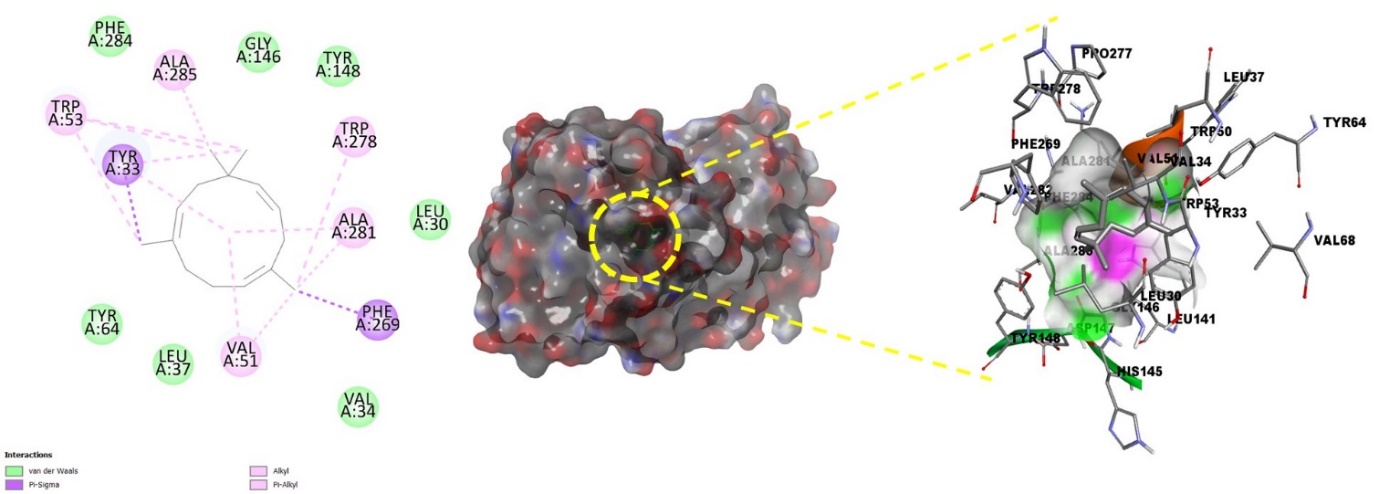
**

**Figure S22. Molecular interaction studies of humulene with mJHBP (PDB ID: 5V13), surface view (Right panel), and 2D (Left panel) interactions.**

**
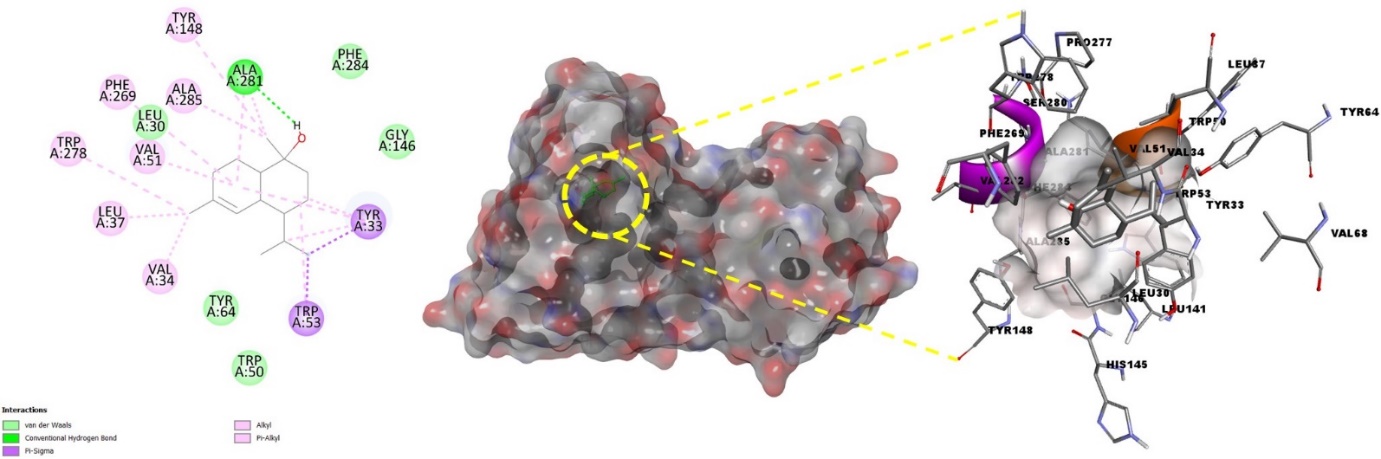
**

**Figure S23. Molecular interaction studies of muurolol with mJHBP (PDB ID: 5V13), surface view (Right panel), and 2D (Left panel) interactions.**

**
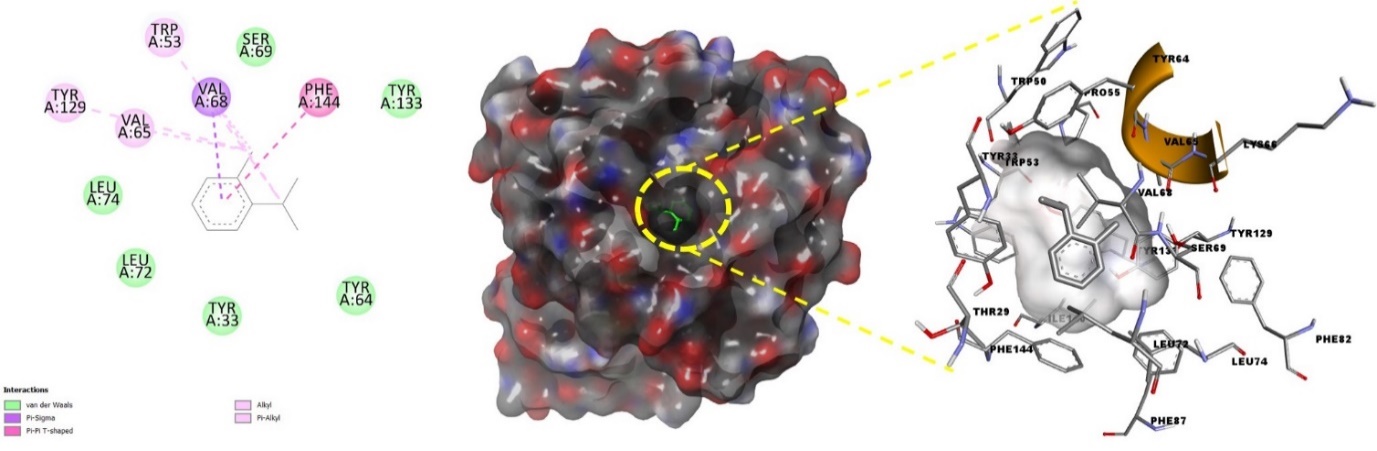
**

**Figure S24. Molecular interaction studies of o-cymene with mJHBP (PDB ID: 5V13), surface view (Right panel), and 2D (Left panel) interactions.**

**
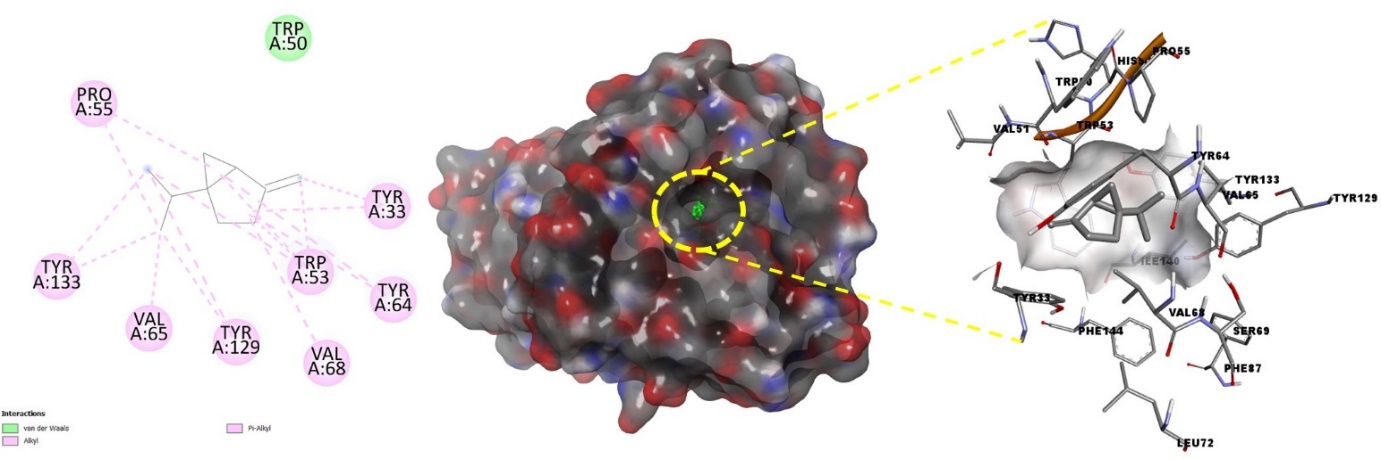
**

**Figure S25. Molecular interaction studies of sabinene with mJHBP (PDB ID: 5V13), surface view (Right panel), and 2D (Left panel) interactions.**

**
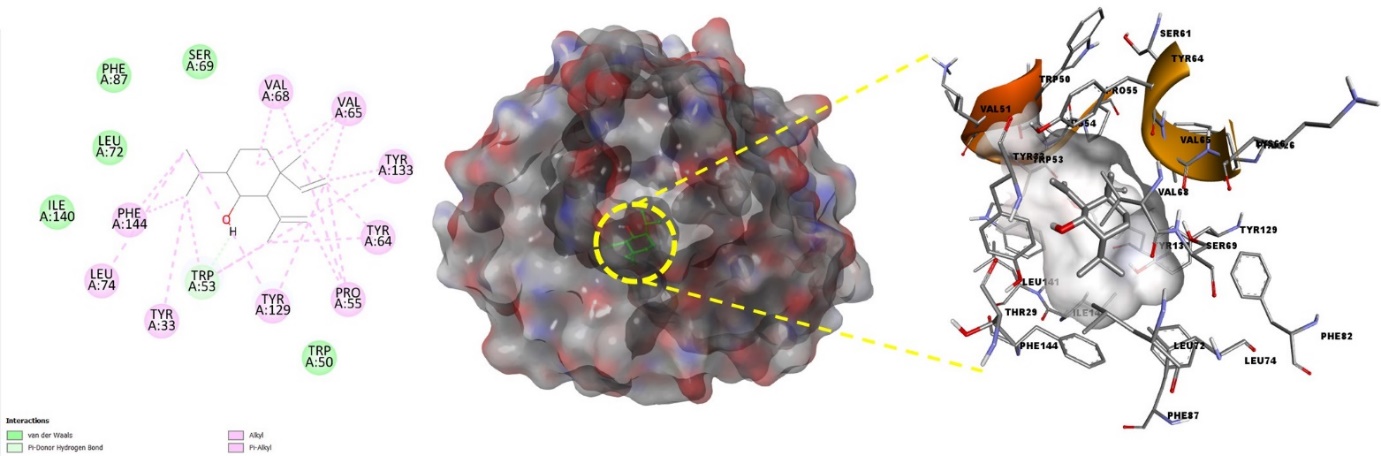
**

**Figure S26. Molecular interaction studies of shyobunol with mJHBP (PDB ID: 5V13), surface view (Right panel), and 2D (Left panel) interactions.**

**
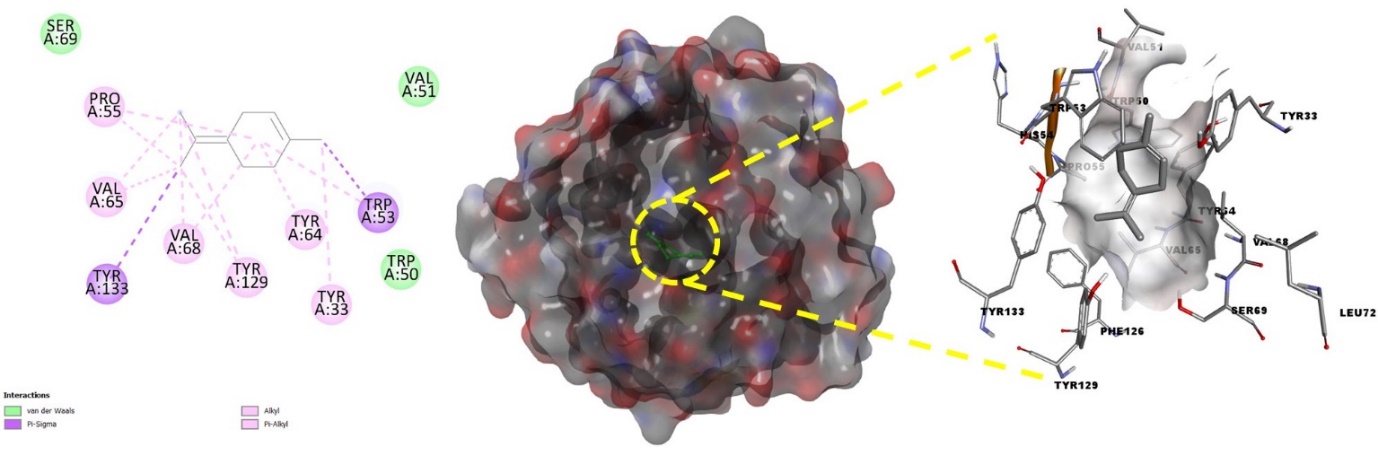
**

**Figure S27. Molecular interaction studies of terpinolene with mJHBP (PDB ID: 5V13), surface view (Right panel), and 2D (Left panel) interactions.**

**
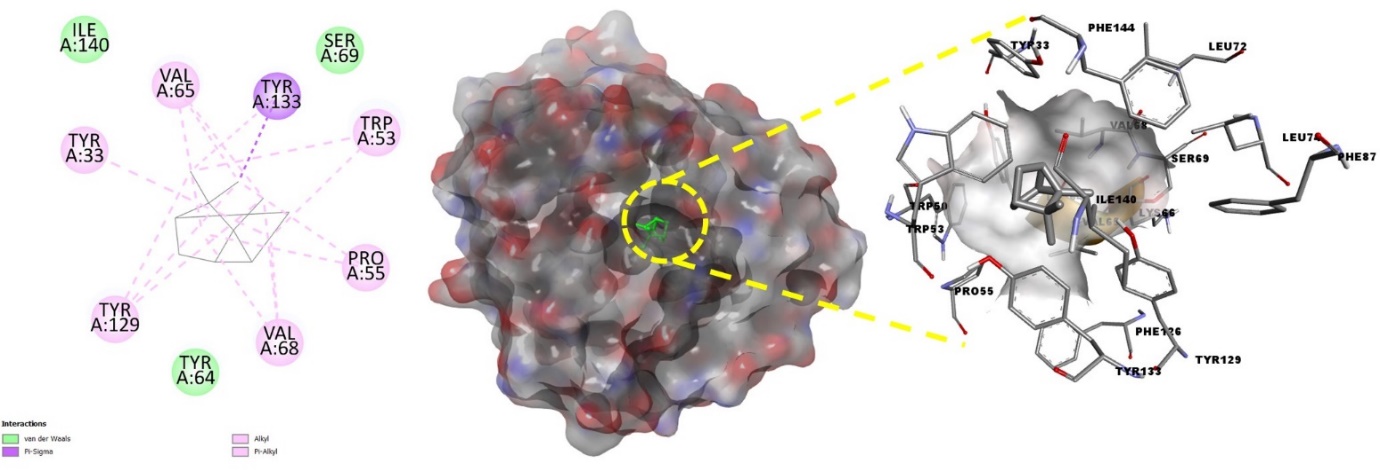
**

**Figure S28. Molecular interaction studies of tricyclene with mJHBP (PDB ID: 5V13), surface view (Right panel), and 2D (Left panel) interactions.**

**
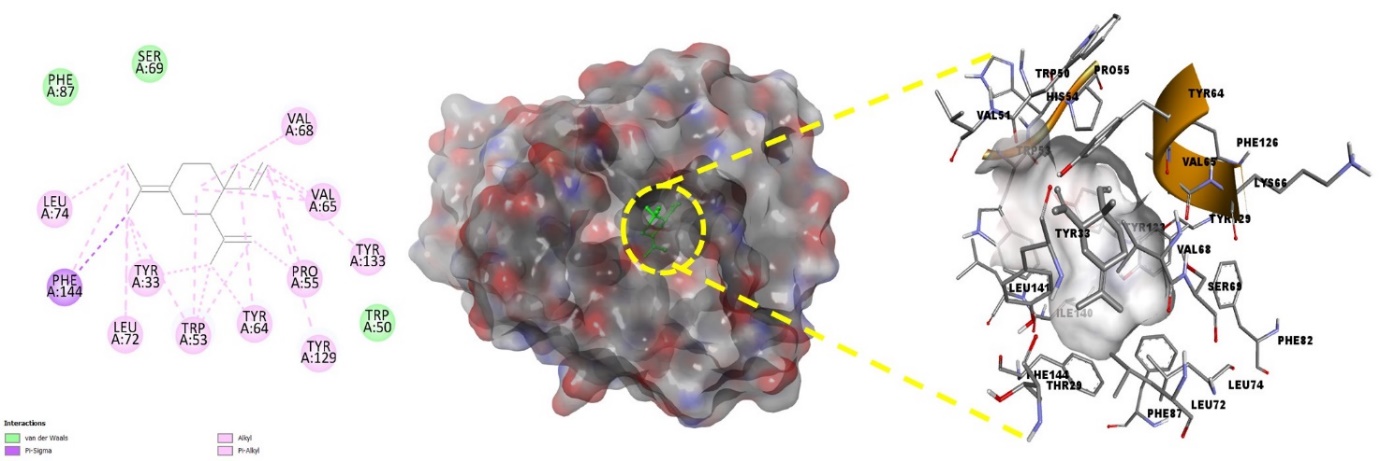
**

**Figure S29. Molecular interaction studies of elixene with mJHBP (PDB ID: 5V13), surface view (Right panel), and 2D (Left panel) interactions.**

**
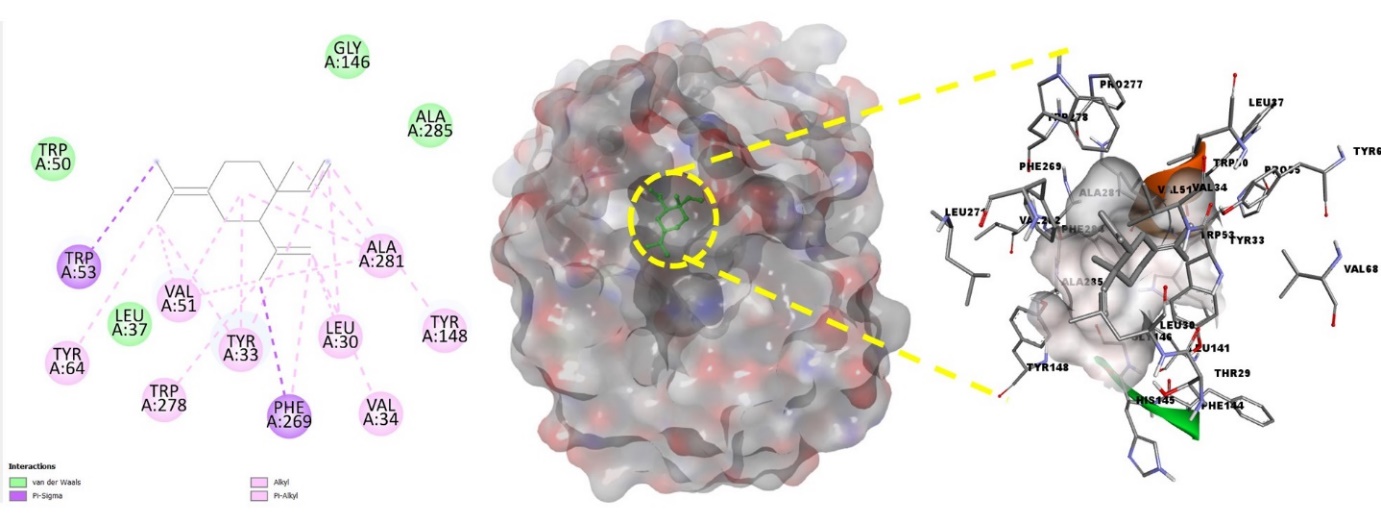
**

**Figure S30. Molecular interaction studies of gamma-elemene with mJHBP (PDB ID: 5V13), surface view (Right panel), and 2D (Left panel) interactions.**

**
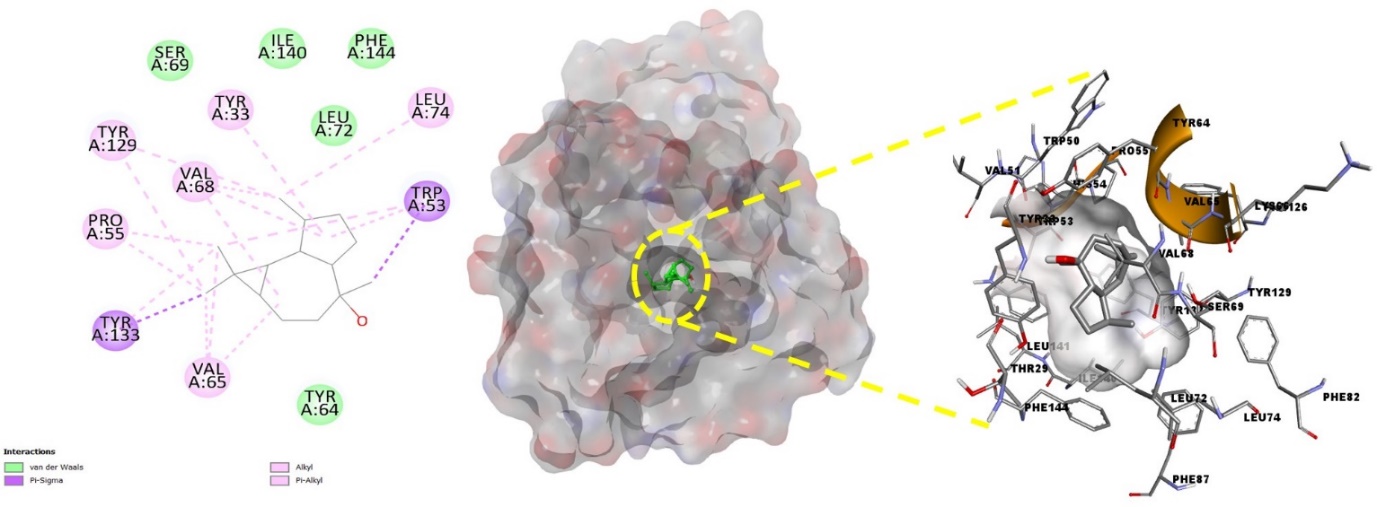
**

**Figure S31. Molecular interaction studies of viridiflorolol with mJHBP (PDB ID: 5V13), surface view (Right panel), and 2D (Left panel) interactions.**


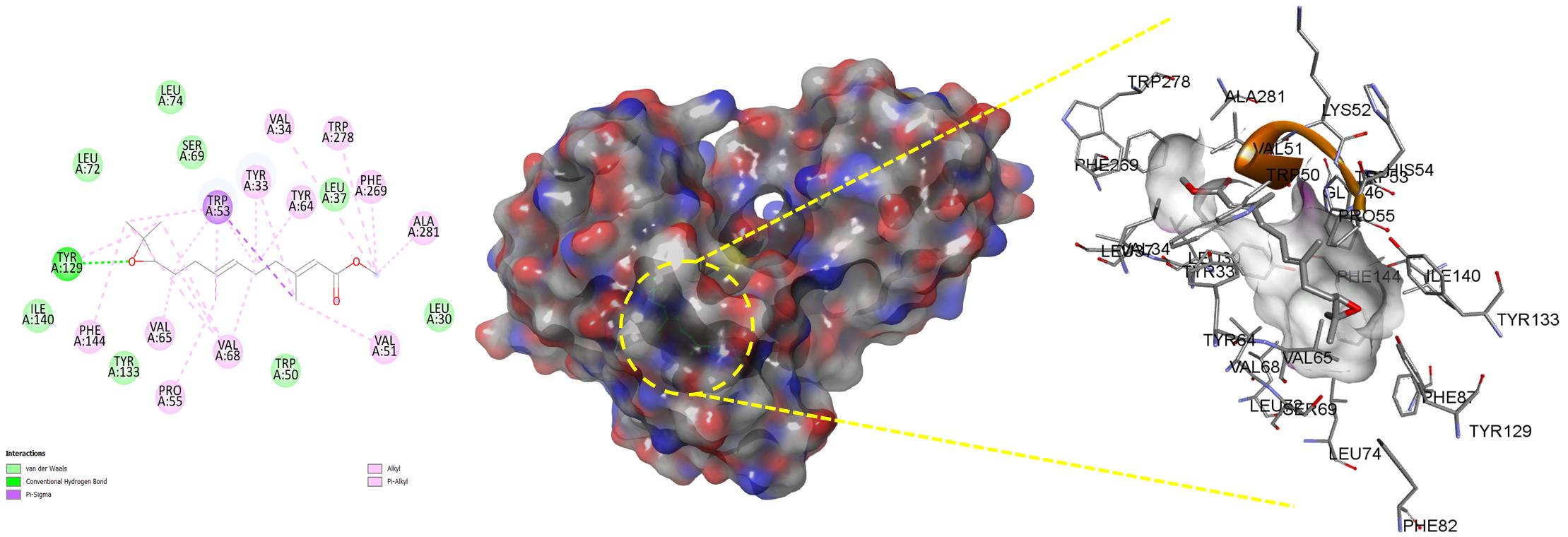


**Figure S32. Molecular interaction studies of JH3 with mJHBP (PDB ID: 5V13), surface view (Right panel), and 2D (Left panel) interactions.**
